# Optimization of microwave components using machine learning and rapid sensitivity analysis

**DOI:** 10.1038/s41598-024-82701-3

**Published:** 2024-12-28

**Authors:** Slawomir Koziel, Anna Pietrenko-Dabrowska

**Affiliations:** 1 Engineering Optimization & Modeling Center, Reykjavik University, 101 Reykjavik, Iceland; 2 Faculty of Electronics, Telecommunications and Informatics, Gdansk University of Technology, Gdansk, 80-233 Poland

**Keywords:** Microwave engineering, CAD, Optimization, Global search, Machine learning, Sensitivity analysis, Behavioral modeling, Nature-inspired algorithms, Engineering, Electrical and electronic engineering

## Abstract

Recent years have witnessed a tremendous popularity growth of optimization methods in high-frequency electronics, including microwave design. With the increasing complexity of passive microwave components, meticulous tuning of their geometry parameters has become imperative to fulfill demands imposed by the diverse application areas. More and more often, achieving the best possible performance requires global optimization. Unfortunately, global search is an intricate undertaking. To begin with, reliable assessment of microwave components involves electromagnetic (EM) analysis entailing significant CPU expenses. On the other hand, the most widely used nature-inspired algorithms require large numbers of system simulations to yield a satisfactory design. The associated costs are impractically high if not prohibitive. The use of available mitigation methods, primarily surrogate-based approaches, is impeded by dimensionality-related problems and the complexity in microwave circuit characteristics. This research introduces a procedure for expedited globalized parameter adjustment of microwave passives. The search process is embedded in a surrogate-assisted machine learning framework that operates in a dimensionality-restricted domain, spanned by the parameter space directions being of importance in terms of their effects on the circuit characteristic variability. These directions are established using a fast global sensitivity analysis procedure developed for this purpose. Domain confinement reduces the cost of surrogate model establishment and improves its predictive power. The global optimization phase is complemented by local tuning. Verification experiments demonstrate the remarkable efficacy of the presented approach and its advantages over the benchmark methods that include machine learning in full-dimensionality space and population-based metaheuristics.

## Introduction

High-frequency system design, in general, and microwave passive component development are more challenging than ever. In large part, this is a result of increasing performance requirements, which reflect the requirements of emerging application areas^1–6^, both in terms of specifications imposed on electrical characteristics (e.g., multi-band operation, harmonic suppression, etc^7–9^). , , and particular functionalities (reconfigurability^10^, unconventional phase characteristics^11^). In a growing number of situations, one of the essential design considerations is miniaturization^12–15^. Maintaining small size of passive components often requires the utilization of transmission line (TL) folding^16^, defected ground structures^17^, miniaturized resonant cells^18^, or metamaterials^19,20^. To meet the strict performance thresholds, microwave circuits become increasingly more complex and parameterized by larger numbers of design variables as compared to conventional architectures. Meticulous tuning of these parameters is imperative to achieve the best possible operation^21^. The latter is an intricate process, not only due to the need simultaneous handling of many variables, design goals, and constraints, but also because accurate assessment of passive circuits necessitates electromagnetic (EM) simulation. Full-wave analysis is necessary to quantify essential phenomena such as cross-coupling, the effect of connectors, and various losses (e.g., dielectric, radiation).

The aforementioned factors make the importance of simulation-based design continuously growing in microwave engineering^22–24^. Yet, it is a challenging undertaking: any numerical procedure entails repetitive system analyses, which generates considerable computational expenses. For example, local (gradient-based) tuning costs from a few dozen for simple circuits to several hundreds of EM analyses for more complex scenarios. Furthermore, proper analytical formulation of the design task may be a non-trivial matter due to the need to handle several goals and constraints. Furthermore, the lack of a decent starting point may result in inferior results. Given these difficulties, many researchers default to interactive methods such as experience-driven parametric studies, which often lead to satisfactory (yet sub-optimal) results but are extremely laborious, thereby affecting time-to-market and the overall efficacy of the design process. Global or multi-criterial optimization generates significantly higher expenses (typically, thousands of EM simulations). On the other hand, global search has been recommended for a growing number of tasks, primarily due to a possible presence of multiple local optima (i.e., problem multimodality). Examples include the design of frequency selective surfaces^25^, metamaterial-based components^26,27^, coding metasurfaces^28^, antenna array pattern synthesis^29,30^, design of sparse^31^ and conformal arrays^32^. Globalized optimization is also necessary if a good initial composition of design variables is unavailable to enable local tuning (e.g., in re-design of filtering or coupling circuits over wide ranges of frequencies^33^ or material parameters^34^) or in the case of parametric redundancy as in the case of miniaturized (especially CMRC-based) components^35^.

Since early 1990s, the most widely employed category of techniques for general-purpose global optimization have been nature-inspired population-based metaheuristic algorithms^36–40^. The operating principle of these methods is to mimic various social^41^ or natural processes^42^, including natural evolution^43^ or preying strategies of animals^44,45^. Rather than processing a single solution, population-based algorithms handle the entire sets of candidates for the optimum design (also referred to as swarms, packs, etc.)^46^; the candidates themselves are named individuals (agents, particles, etc.)^47^. The global search ability arguably results from exchanging data within the population, which may directly affect their composition (e.g., by means of recombination operators^48^) or relocation (by biasing it towards individual/global best solution^49^). An important role is played by stochastic components introduced in various forms, e.g., randomized selection procedures^50^, mutation operators based on chosen probability distributions^51^, etc. A common drawback of nature-inspired algorithms is their tremendous computational cost, typically equivalent to thousands of merit function evaluations. Also, the solution repeatability is often mediocre, largely depending on available computational budgets. The discussed category of methods contains a large number of specific algorithms. The popular ones are genetic/evolutionary algorithms, genetic programming, ant systems^52–55^, particle swarm optimizers (PSO)^56^, differential evolution (DE)^57^, firefly algorithm^58^, or harmony search^59^. In fact, there are numerous new methods developed every year^60–64^; however, the differences between them are minor. One of the sources of the popularity of nature-inspired techniques is easy implementation and handling. Unfortunately, the mentioned poor computational efficiency makes these methods unsuitable for direct EM-driven design, unless individual simulation time is short (say, 10–30 s). Not surprisingly, in high-frequency electronics, population-based algorithms are primarily used if analytical models are available. A flagship example is pattern optimization of antenna arrays with the help of analytical array factor models^65,66^.

The difficulties associated with the high cost entailed by massive EM analyses needed in nature-inspired methods may be partially alleviated using surrogate modeling techniques^67–69^. Practical procedures, often referred to as machine learning frameworks^70–72^, operate iteratively with a fast metamodel (kriging, neural networks^73–76^) employed to predict subsequent approximations of the optimum design while being gradually refined based on the EM data acquired in the process^77^. The supplementary points may be generated using various infill criteria either oriented towards the improvement of the surrogate model accuracy (space exploration^78^) or identification of the optimum design (space exploitation^79^). While surrogate-assisted methods offer advantages over direct EM-driven design, their fundamental bottleneck is the construction of a reliable metamodel. It is impeded by two main factors, one being the curse of dimensionality and the other structural complexity of circuit outputs, both as a function of geometry variables and frequency. In the literature, many techniques of this class are therefore demonstrated using rather simple test cases defined over low-dimensionality parameter spaces^80–82^. Some recent approaches (e.g^122–124^), demonstrated improved handling of problem dimensionality through a smart combination of artificial-intelligence (AI)-based tools. Addressing the mentioned difficulty has encouraged extensive research efforts. Some of the available mitigation techniques include performance-driven modeling^83–86^, variable-fidelity approaches^87–89^, as well as the response feature methods^90–93^. In the realm of physics-based models, we have space mapping^94^, cognition-driven design^95^, and a variety of response correction methods^96,97^. Unfortunately, none of the mentioned approaches is generic; for example, the applicability of feature-based optimization is contingent upon the existence of characteristic points selected for the system of interest across the complete design variable space^98^. On the other hand, space mapping requires the existence of a sufficiently accurate lower-fidelity representation.

This research introduces an innovative algorithm to enhanced-efficacy global parameter adjustment of microwave circuits. Our methodology belongs to a category of machine learning procedures and employs the (globalized) merit function enhancement as the criterion for generating supplementary candidate designs. The core optimization engine is PSO, whereas the underlying metamodel is produced by means of kriging. The key acceleration mechanism is to conduct the search process over a dimensionality-restricted domain, determined based on a small number (typically, three to five) of principal vectors associated with the maximum variations of the circuit characteristics. These vectors are obtained through fast global sensitivity analysis (GSA) based on a handful of random samples as well as the spectral analysis of the circuit response variations estimated using the closest neighbors of the respective data points. Unlike conventional GSA techniques, the proposed method employs considerably smaller datasets, and allows for identification or arbitrarily oriented vectors (i.e., not necessarily coinciding with the coordinate system axes), thereby ensuring improved flexibility. Establishing surrogate models in the confined domain enables lower running expenses of the parameter adjustment process. The accuracy loss due to dimensionality reduction is compensated for by local tuning of the circuit under design. The proposed framework is comprehensively verified based on four microstrip circuits. Comparisons with direct nature-inspired optimization, multiple-start gradient search, and a machine learning framework operating in the full-dimensionality space, underscore the competitive performance of the proposed method. The benefits pertain to reliability, design quality, and computational efficiency. The average expenses incurred by the optimization algorithm correspond to just 200 EM simulations of the circuit, which makes our technique a potentially interesting alternative to optimization frameworks available in the literature.

## Global optimization using dimensionality-reduced surrogates and machine learning

Here, we explain the main components of the global optimization strategy introduced in the paper. The foundation of the approach is dimensionality reduction realized using fast global sensitivity analysis (FGSA). FGSA identifies the essential directions within the parameter space based on their impact on circuit response variability. The sub-space defined by these influential directions serves as the domain for the data-driven replacement model, which is constructed with a training dataset of a limited cardinality. The core optimization engine is employs machine learning (ML) principles, using dimensionality-reduced kriging surrogates as predictors and a PSO as the core search routine. The ML search process utilizes the improvement of the cost function to generate the candidate designs. The final design is achieved through auxiliary local parameter tuning.

The remaining part of this section is arranged as elucidated below. Section 2.1 recalls the mathematically rigorous formulation of the EM-driven optimization task. Section 2.2 describes the fast global sensitivity analysis procedure. Section 2.3 elaborates on the global search stage, whereas Sect. 2.4 discusses the local tuning algorithm. Section 2.5 puts together the complete procedure.

### Simulation-driven design of microwave circuit. Problem formulation

**Fig. 1:**
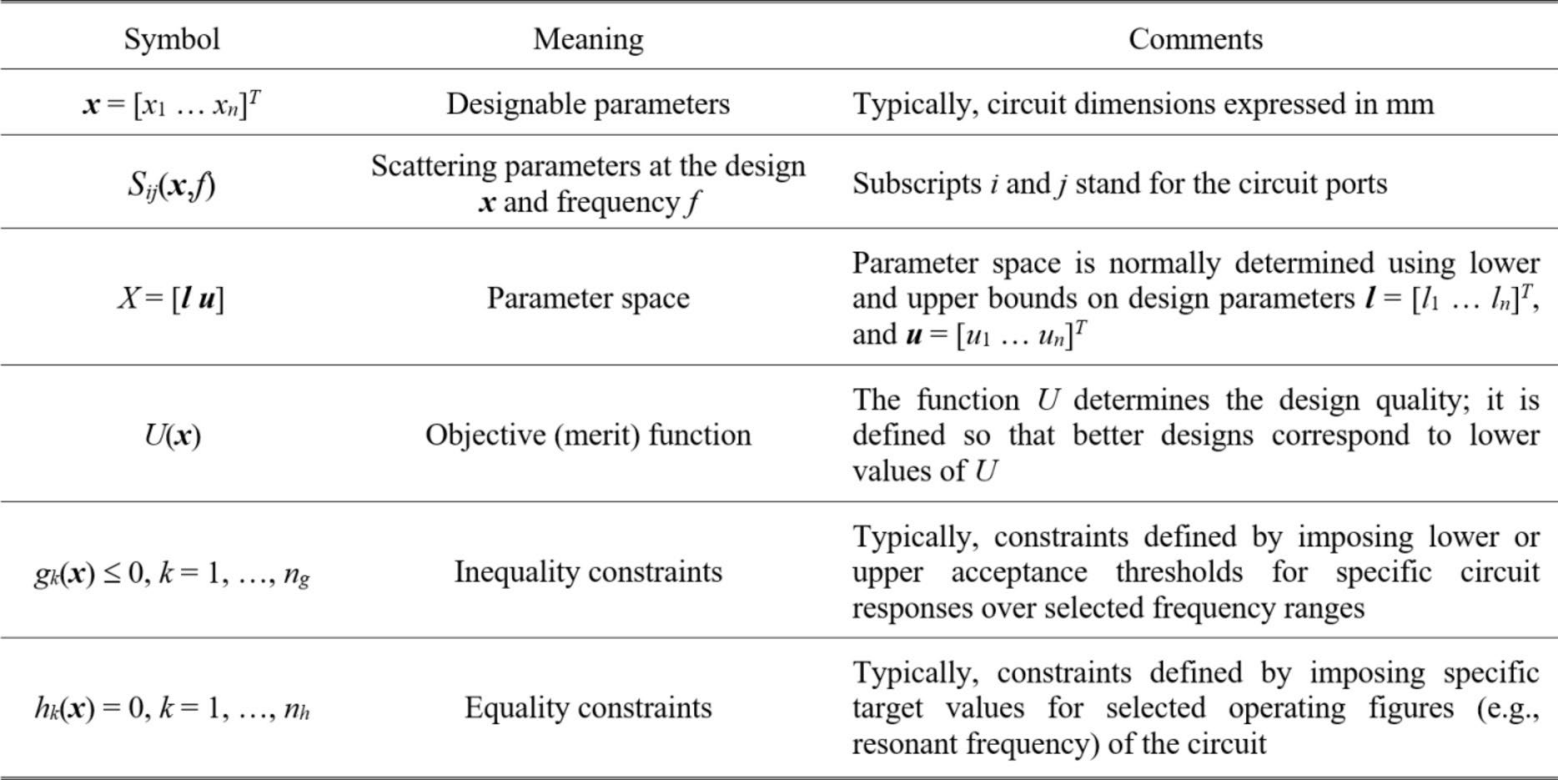
EM-based design of microwave components: notation and terminology.

Achieving optimal performance in passive microwave components requires precise tuning of their geometry parameters. In this work, the design variables are organized into a vector ***x***, as depicted in Fig. 1, which also introduces the notation used for simulation-driven design. The design variable *X*, where the search process occurs, is typically delimited by the lower and upper bounds of the parameters. The quality of design is assessed with the help of a cost function *U*, which varies depending on the specific problem. The primary responses of interest for microwave components are the scattering parameters (as shown in Fig. 1) and various derived quantities such as the phase response. These parameters are evaluated through full-wave EM simulation.

Using the notation of Fig. 1, the parameter tuning task may be stated in the form

1 $${\mathbf{x}^*}=\arg \mathop {{\min} }\limits_{{\mathbf{x} \in X}} U\left( \mathbf{x} \right)$$

where x^*^ denotes the best-performance design we seek for.

Most practical problems are inherently multicriterial. That is, we have more than one objective to improve or control. However, the majority of existing algorithms are single-objective. Consequently, it is necessary to re-state multicriterial tasks to fit to single-objective setups. One possible way is to decide upon a main objective and to control the others as constraints^99^. Another option is scalarization (e.g., a weighted sum method^100^). Direct multi-objective search^101^ is a topic outside this study’s scope of interest.

**Fig. 2:**
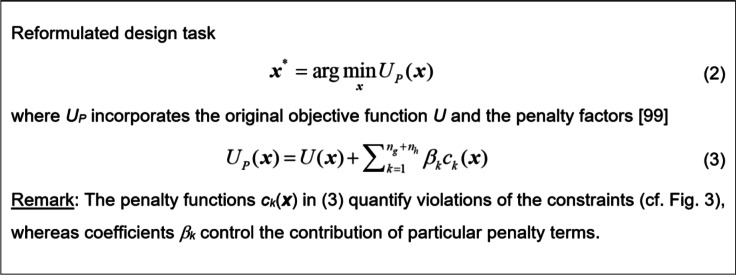
A concept of implicit constraint handling.

In many practical cases, task (1) may be constrained, with either inequality or equality conditions *g*_*k*_(***x***) ≤ 0 and *h*_*k*_(***x***) = 0, respectively (cf. Fig. 1). One of the possible handling methods, especially convenient for computationally expensive conditions *g*_*k*_ and/or *h*_*k*_, is a penalty function approach^99^, briefly recalled in Fig. 2.

**Fig. 3:**
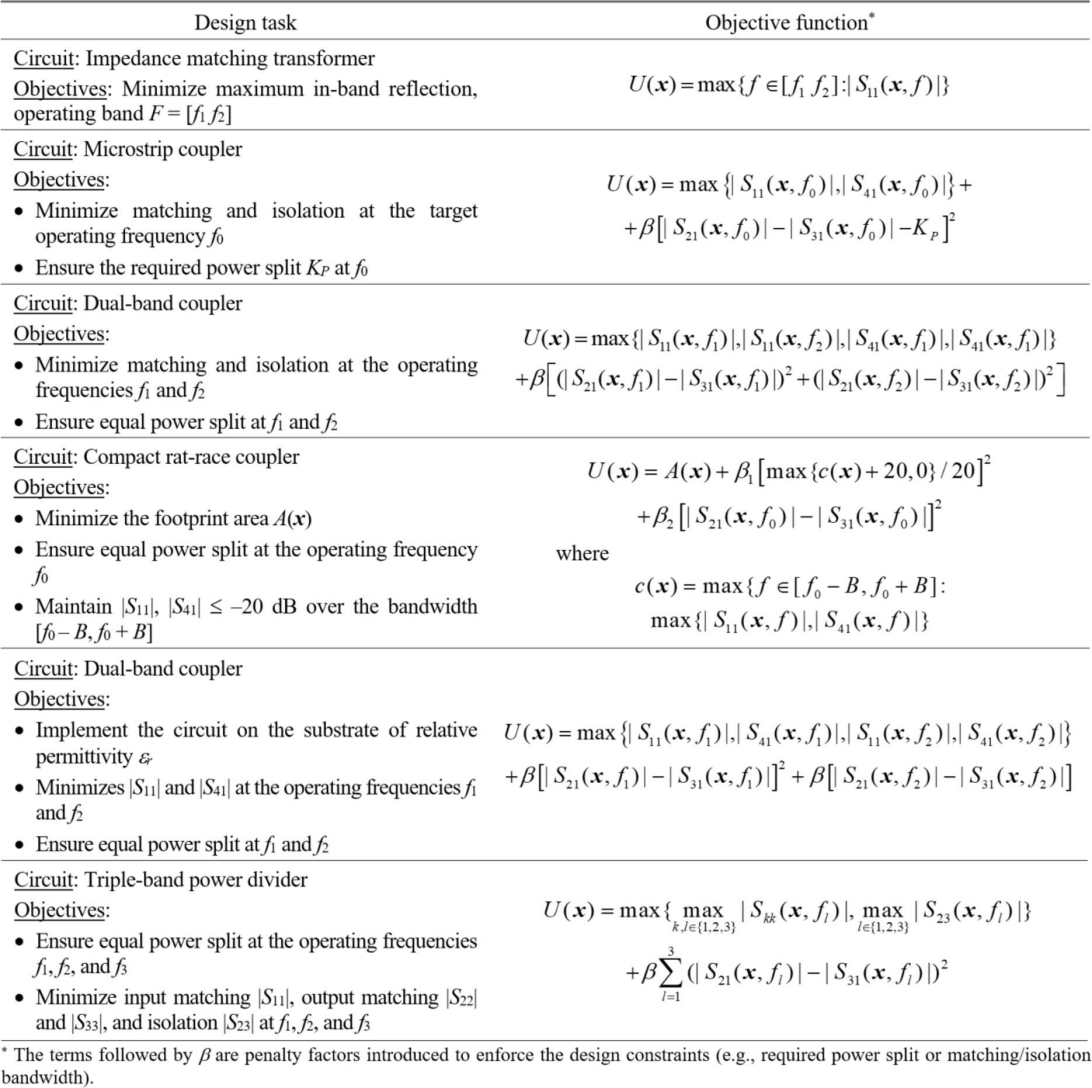
Examples of simulation-driven optimization problems. Task description (left) is accompanied by a possible objective function statement (right).

Figure 3 provides several cases of simulation-based microwave design problems. The corresponding merit functions are defined using implicit constraint handling, and incorporate appropriate penalty terms, following the formulation of Fig. 2. The penalty terms utilize second power [.]^2^ to make sure that the merit function is differentiable regarding violation of the respective constraints. The latter facilitates the search the feasible region boundary, which is important because one or more constraints are active at x^*^.

### Fast global sensitivity analysis

The optimization strategy considered in this work employs data-driven surrogate models. As mentioned earlier, the modelling of microwave components is impeded by several issues, including those related to dimensionality of the design space, circuit output nonlinearity, and a large extent of frequency and geometry/material parameters over which the model must cover to make is suitable for design purposes. The ability to restrict the dimensionality is perhaps the most important factor that enables building reliable metamodels at practically acceptable computational expenses.

Identification of parameters that are of minor importance for the problem at hand (and, therefore, can be excluded) can be realized using variable screening (e.g., Pearson correlation coefficients^102^, partial correlation coefficients^103^, Morris method^104^), or global sensitivity analysis (GSA). Some of commonly used GSA procedures include Sobol indices^105^, regression-based methods^106^, or Jansen method^107^. Unfortunately, most of these techniques require large sets of data samples to evaluate the sensitivity. On the other hand, the dependence between circuit dimensions and electrical characteristics is often quite intricate for microwave components, so the exclusion of individual variables may not be a good option.

Here, we suggest an alternative GSA methodology, which is developed to fulfil the following prerequisites:

- It should be cheap to evaluate, e.g., incur the cost of less than a hundred of EM circuit’s simulations;
- It should lead to identifying important parameter space directions (i.e., those having major effects on electrical characteristic variability), rather than individual parameters (i.e., directions aligned with the coordinate system axes).

The following sub-sections provide the details of the fast GSA (FGSA) procedure developed under the above conditions (Sect. 2.2.1), discuss illustration examples (Sect. 2.2.2), and the determination of a reduced-dimensionality domain established using the principal vectors found by FGSA.

####  Fast sensitivity analysis: formulation

**Fig. 4:**
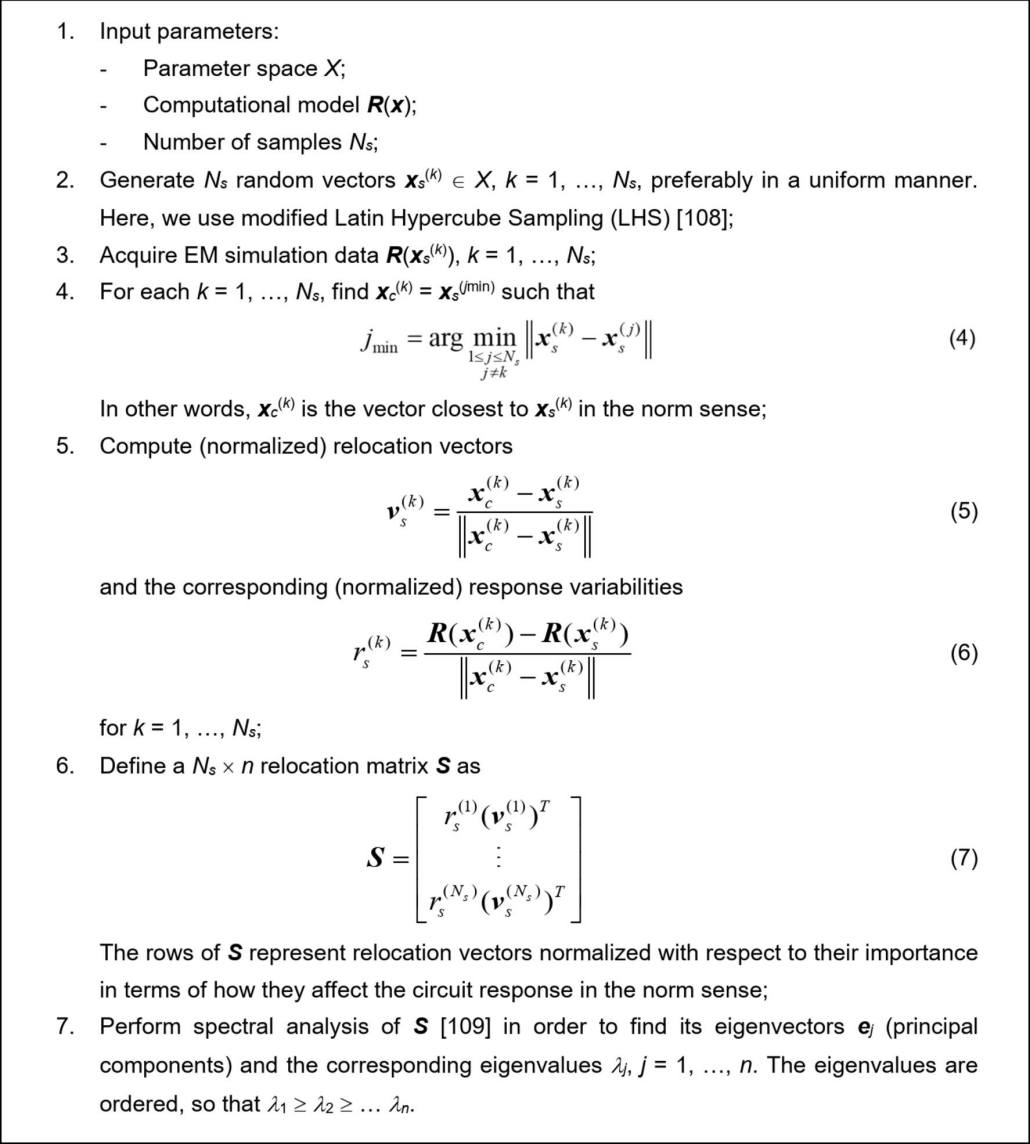
Pseudocode of the fast global sensitivity analysis (FGSA). The eigenvectors ***e***_*j*_ are the vectors having the major effect on the circuit responses; the importance is quantified using the eigenvalues *λ*_*j*_.

The operating details of the fast global sensitivity analysis (FGSA) procedure have been shown in Fig. 4. The key stage of FGSA is the spectral analysis of the relocation matrix ***S***, which produces the eigenvectors ***e***_*j*_ and the corresponding eigenvalues *λ*_*j*_. The former represent the essential directions in the design variable space. The latter, indexed in a descending order, quantify the effects of particular directions on the circuit response variability. Due to being eigenvectors, ***e***_*j*_, *j* = 1, …, *n*, compose an orthonormal basis in the design variable space *X*, i.e., ||***e***_*j*_|| = 1 for *j* = 1, …, *n*, and ***e***_*j*_^*T*^***e***_*k*_ = 0, for *j* ≠ *k*.

A small number *N*_*d*_ < *n* of the most significant directions is used to span the reduced-dimensionality domain for the global search stage described in Sect. 2.3. More specifically, *N*_*d*_ ∈ {1, 2, …, *n*} is the smallest integer for which

8 $$\frac{{\sqrt {\sum\nolimits_{{j=1}}^{{{N_d}}} {\lambda _{j}^{2}} } }}{{\sqrt {\sum\nolimits_{{j=1}}^{n} {\lambda _{j}^{2}} } }} \geqslant {C_{{\min} }}$$

Here, *C*_min_ is a user-defined threshold (a user-defined control threshold). In this study, it is set to *C*_min_ = 0.9. According to (8), *N*_*d*_ is the number of eigenvectors for which the overall (relative) least-square response variability exceeds *C*_min_, which is 90% for the mentioned value of 0.9.

#### Examples

**Fig. 5:**
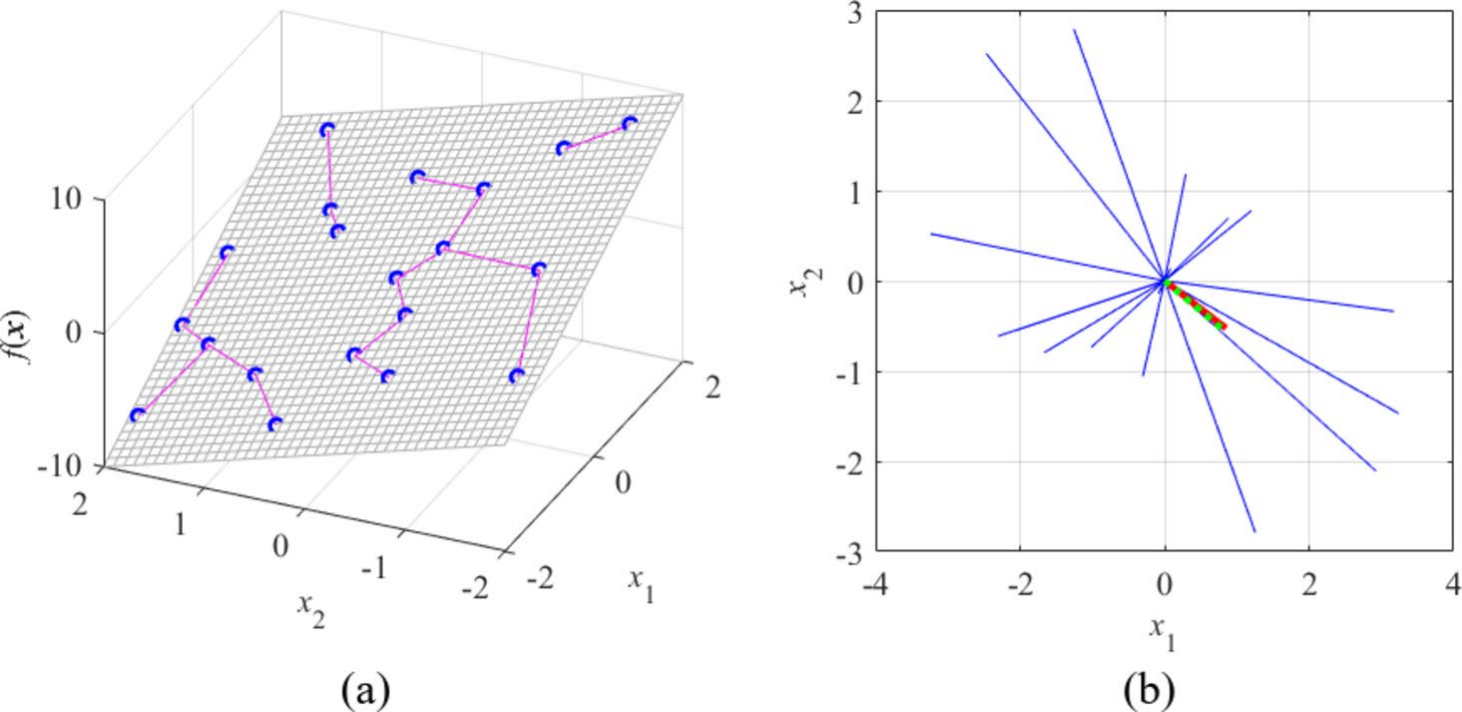
FGSA illustration using a linear function *f*(***x***) = *f*([*x*_1_* × *_2_]^*T*^) = 3* × *_1_ – 2* × *_2_: (**a**) surface plot of the function (gray), twenty random observables ***x***_*s*_^(*k*)^ (circles), and relocation vectors ***x***_*c*_^(*k*)^ – ***x***_*s*_^(*k*)^ (line segments); (**b**) relocation matrix vectors *r*_*s*_^(*k*)^***v***_*s*_^(*k*)^ (thin lines), the largest principal component ***e***_1_ (thick solid line), and the normalized gradient ***g*** = [3 − 2]^*T*^/13^1/2^ (thick dotted line). In this example, all function variability occurs along the gradient ***g*** (the function is constant in the direction orthogonal to ***g***), which is well aligned with the vector ***e***_1_, obtained using the proposed FGSA.

In this section, FGSA is illustrated in several analytical examples and a microwave coupler circuit. The first case is a simple linear function *f*(***x***) = *f*([*x*_1_* × *_2_]^*T*^) = 3* × *_1_ – 2* × *_2_, cf. Fig. 5. The function *f* is intentionally chosen as linear, allowing us to unanimously determine the maximum variability direction, i.e., the gradient vector ***g*** = [3 − 2]^*T*^. The FGSA procedure has been carried out using twenty random observables, leading to the same result (cf. Fig. 5b).

**Fig. 6:**
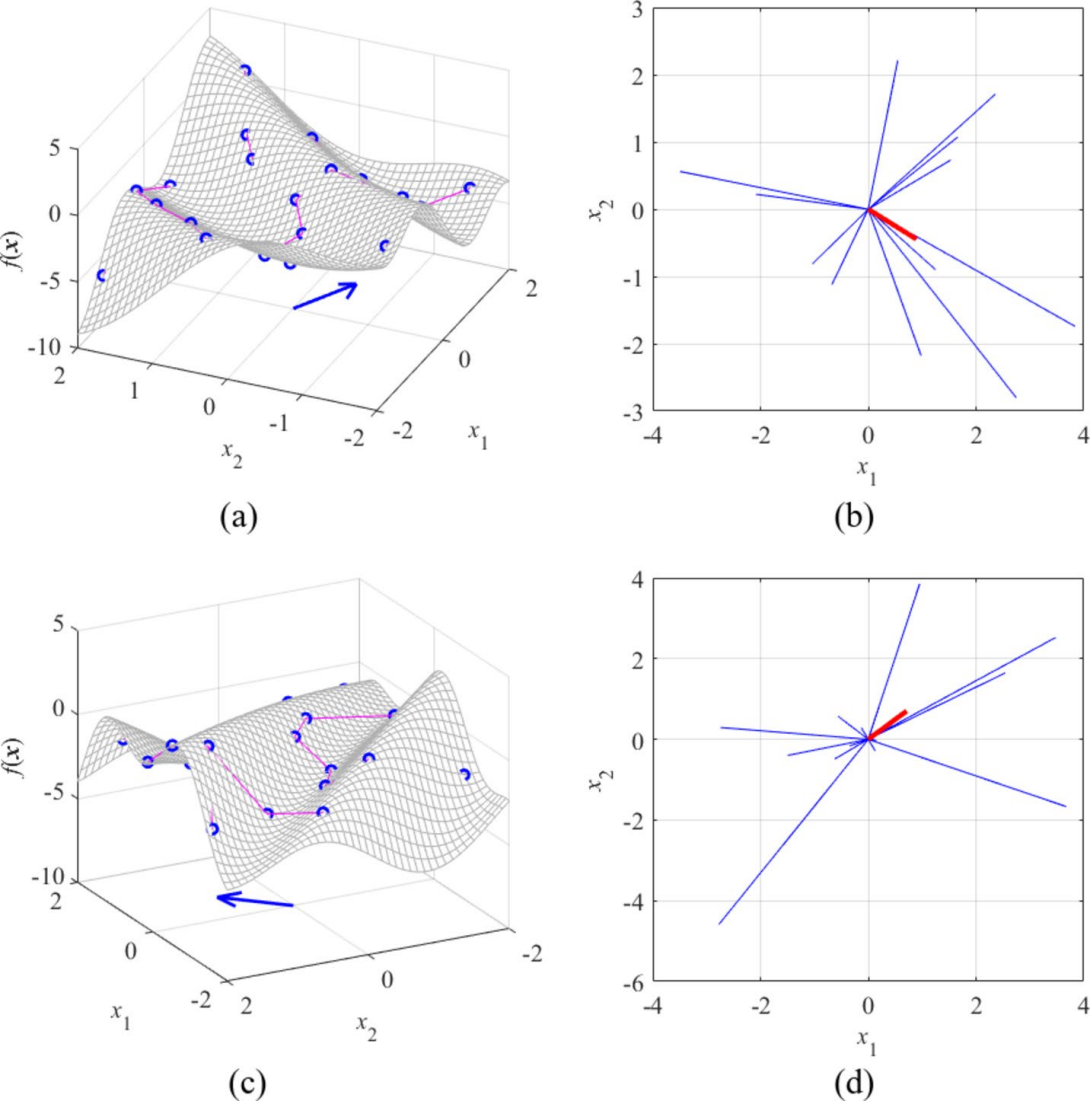
FGSA illustration using nonlinear functions of two variables: (**a**) surface plot of the first function (gray), twenty random observables ***x***_*s*_^(*k*)^ (circles), and relocation vectors ***x***_*c*_^(*k*)^ – ***x***_*s*_^(*k*)^ (line segments), as well as the principal component ***e***_1_ (thick arrow); (**b**) relocation matrix vectors *r*_*s*_^(*k*)^***v***_*s*_^(*k*)^ (thin lines), and the largest principal component ***e***_1_ (thick solid line); (**c**) and (**d**) surface plot and relocation matrix vectors for the second function. It can be noticed that the vector ***e***_1_ obtained using FGSA visually corresponds to the direction of the largest variability of *f*(***x***).

Figure 6 shows two further examples. In both cases, the respective functions are defined to permit visual assessment of the maximum variability direction, which is perpendicular to the function ‘waves.’ Again, this has been confirmed by FGSA, also performed using twenty random samples.

**Fig. 7:**
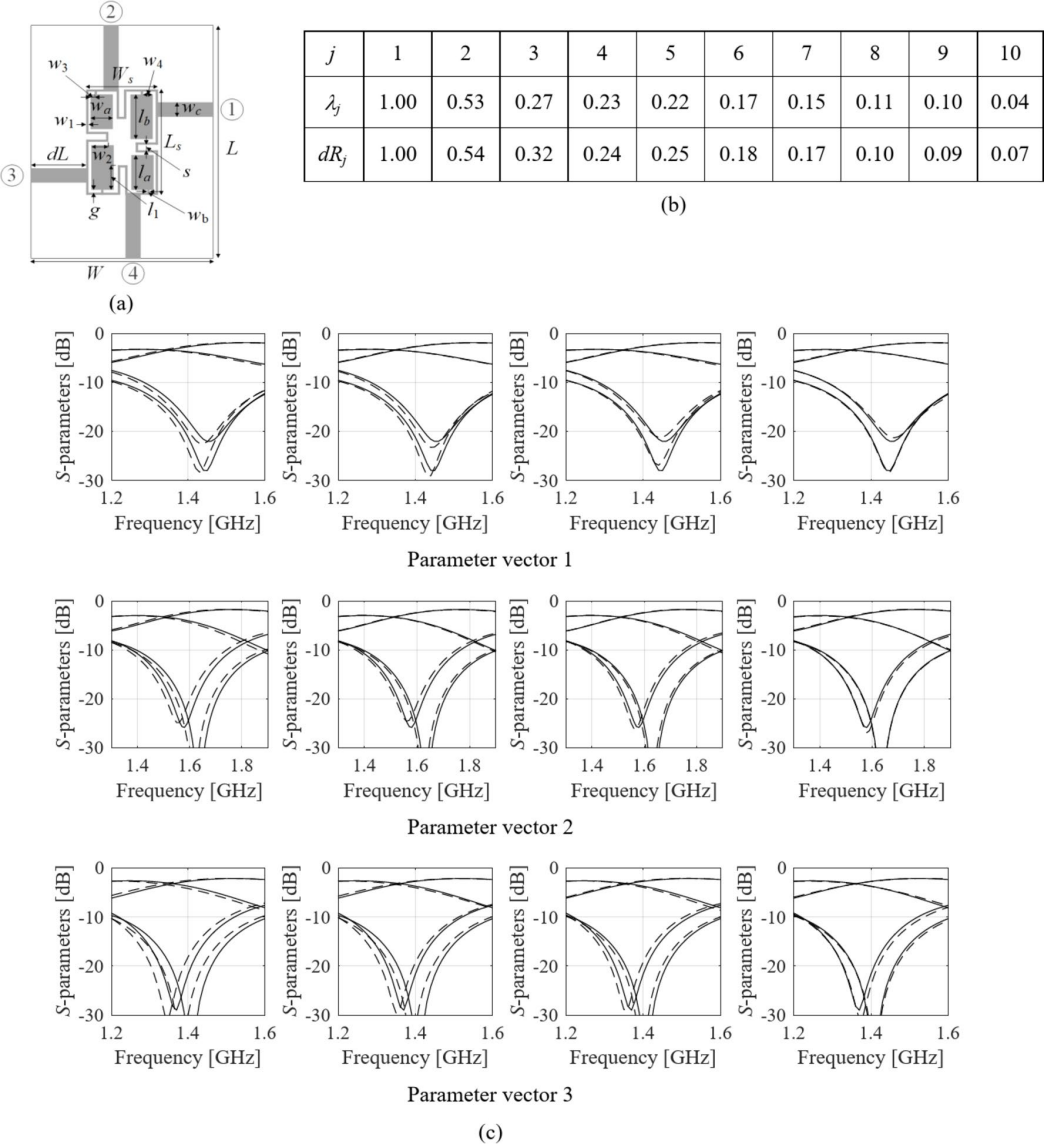
FGSA illustration using a miniaturized branch-line coupler: (**a**) parameterized coupler geometry; (**b**) normalized eigenvalues of the relocation matrix ***S*** obtained using FSGA based on fifty random samples, as well as average EM-simulated variability indicators *dR*_*j*_ computed as in (9); (**c**) *S*-parameters at three random designs (top, middle, and bottom panels), and designs perturbed along the first four principal components, ***x*** + *h****e***_*k*_ (here, *h* = 0.1) (from left to right) obtained using FGSA, Note that response variability is gradually reduced for increasing *k*, which demonstrates that subsequent eigenvectors correspond to directions having less and less effect on the circuit characteristics.

The final illustration case is a compact CMRC-based equal-split coupler. The structure’s architecture has been shown in Fig. 7a. There are ten parameters, ***x*** = [*g l*_1*r*_*l*_*a*_*l*_*b*_*w*_1_*w*_2*r*_*w*_3*r*_*w*_4*r*_*w*_*a*_*w*_*b*_]^*T*^. The design variable space *X* is defined by the parameter bounds ***l*** = [0.4 0.6 3.0 9.0 0.6 0.4 0.1 0.6 4.0 0.6]^*T*^, and ***u*** = [0.5 0.9 6.5 11.0 0.95 0.7 0.4 0.9 5.0 0.9]^*T*^. FGSA has been carried out using fifty random samples allocated uniformly in *X*.

Figure 7c illustrates the coupler’s *S*-parameters at random parameter vector ***x***^(*j*)^, *j* = 1, …, 4, and designs perturbed along the eigenvectors ***e***_*k*_, i.e., ***x***^(*j*)^ + *h****e***_*k*_, *k* = 1, …, *n*. As it can be observed, the average response variability is the most significant for *k* = 1; it is then reduced for increasing *k*, as expected based on the eigenvalue analysis (cf. Fig. 7b.

To obtain numerical data, the actual EM-simulated variability of the *S*-parameters has been evaluated based on *N*_*r*_ = 50 random designs, ***x***_*r*_^(*k*)^, *k* = 1, …., *N*_*r*_, and the corresponding perturbations ***x***_*r*_^(*k.j*)^ = ***x***_*r*_^(*k*)^ + *h****e***_*j*_, *j* = 1, …, *n*. For notational simplicity, ***R***(***x***) will be employed to denote the aggregated circuit response, here, *S*_*k*1_(***x***,*f*) for *k* = 1, 2, 3, 4, obtained for a discrete set of frequencies *f* ∈ {*f*_1_, …, *f*_*m*_}. Using ***R***(***x***_*r*_^(*k*)^), *k* = 1, …, *N*_*r*_, and ***R***(***x***_*r*_^(*k.j*)^), *k* ∈ {1, …, *N*_*r*_}, *j* ∈ {1, …, *n*}, the response variability factors are calculated as

9 $$d{R_j}=\frac{1}{N}{\sum\limits_{{k=1}}^{{{N_r}}} {\left\| {{{\varvec{R}}_f}\left( {x_{r}^{{(k)}}} \right) - {R_f}\left( {x_{r}^{{(k.j)}}} \right)} \right\|} ^2}$$

for *j* = 1, …, *n*. According to (9), *dR*_*j*_ represents the average response variability associated with the direction ***e***_*j*_. As demonstrated in Fig. 7b, the normalized values of *dR*_*j*_ are in reasonable agreement with the normalized eigenvalues *λ*_*j*_, which corroborates the relevance of FGSA.

The FGSA procedure has been developed having in mind computational efficiency as well as flexibility. As it involves a handful of random samples, the FGSA cost is significantly lower than that of conventional sensitivity analysis methods (e.g., regression-based techniques^106^, Sobol indices^105^), even though the evaluation accuracy is somewhat compromised. On the other hand, the presented approach allows for identifying arbitrarily oriented principal directions, which do not have to be coincide with the coordinate system axes. As a result, it permits the exploration of variable interactions instead of eliminating individual circuit dimensions.

#### Model domain definition

The FGSA procedure is employed to determine the most relevant directions within the design variable space *X*. The importance is quantified concerning their effects on the circuit response variations. A subset thereof, namely *N*_*d*_ eigenvectors ***e***_*j*_, *j* = 1, …, *N*_*d*_, are employed to define a dimensionality-reduced set *X*_*d*_, which will be a region of interest for the first optimization phase, which is global search. In particular, *X*_*d*_ will be a domain for the surrogate model to be used within the machine learning framework described in Sect. 2.3.

**Fig. 8:**
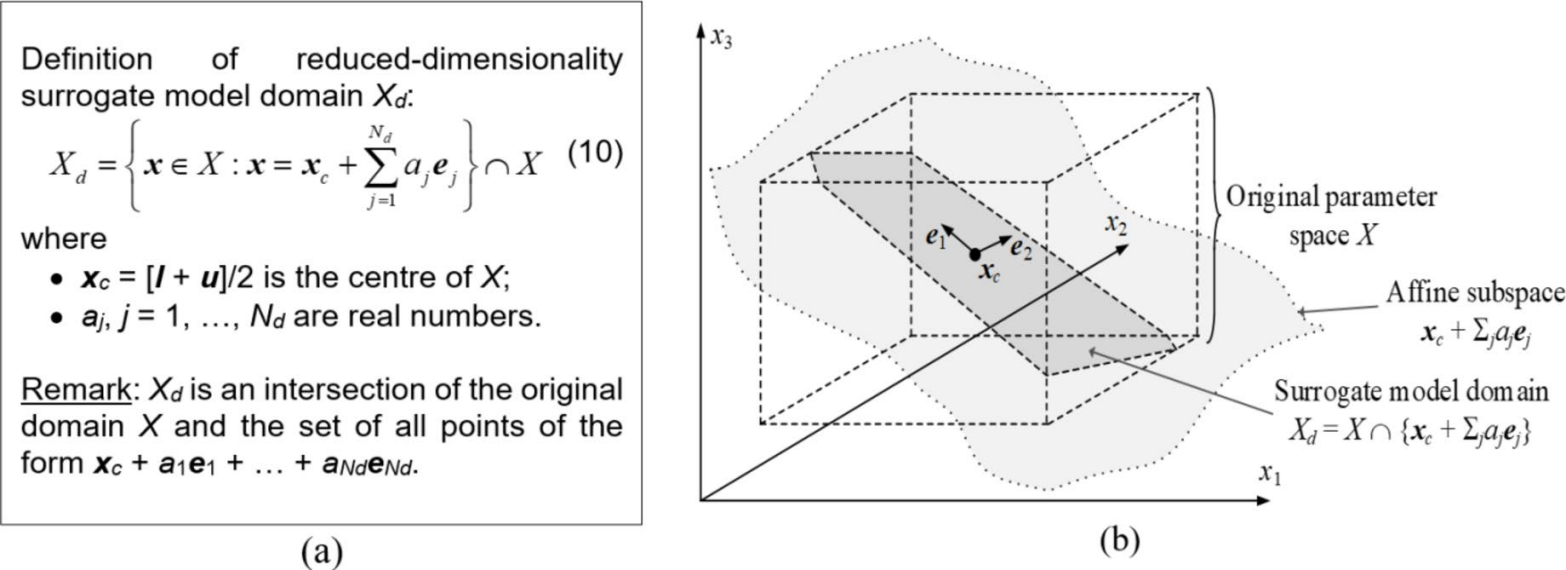
Model domain determined by means of FGSA: (**a**) formal definition, (**b**) graphical illustration of the dimensionality-restricted domain *X*_*d*_. Here, the original parameter space is three-dimensional, whereas the set Xd is established using two principal components, ***e***_1_ and ***e***_2_. It should be emphasized that *X*_*d*_ is a set theory intersection of *X* and the affine subspace ***x***_*c*_ + Σ_*j*=1,2_*a*_*j*_***e***_*j*_.

A formal definition of *X*_*d*_ can be found in Fig. 8a. Figure 8b conceptually illustrates the domain construction process. Note that the domain is essentially a set-theory intersection of the original space *X* and an affine subspace spanned by the first *N*_*d*_ eigenvectors and shifted to the center vector ***x***_*c*_. Dimensionality reduction is instrumental in constructing a reliable surrogate model. As the original space *X* is typically large (i.e., featuring large upper-to-lower bound ratios), establishing a metamodel is extremely difficult and requires large numbers of data samples. As *N*_*d*_ is considerably smaller than *n* (typically, 0.3*n* ≤ *N*_*d*_ ≤ 0.4*n*, cf. Section 3), usable metamodel may be rendered based on a relatively small amount of training data, which carries over into lower cost of the procedure. Meanwhile, *X*_*d*_ accounts for the directions having the major effects on variability of circuit’s frequency characteristics, which secures its design utility.

### Optimization process. Stage I: global search

The initial stage of the search process is global optimization. It is carried out within the domain *X*_*d*_ (cf. Section 2.2.3), and begins by building an initial surrogate model, followed by a machine learning process oriented towards identification of the global optimum. The latter is iterative, with the PSO algorithm generating subsequent candidate designs (infill points). Using this additional data, the surrogate model is refined after completing each iteration. The new points are allocated as the surrogate-predicted cost function minima. Both steps, the initial surrogate construction, and the machine learning routine, are elaborated on in Sect. 2.3.1 and 2.3.2, respectively.

#### Initial surrogate

The first (or initial) data-driven model is rendered using kriging^110^ in the reduced-dimensionality set *X*_*d*_. We used *N*_*i*_*N*_*d*_ training samples, where *N*_*i*_ is a control parameter (here, *N*_*i*_ = 20). The data points are marked as ***x***_*B*_^(*k*)^, *k* = 1, …, *N*_*i*_*N*_*d*_. They are uniformly distributed in *X*_*d*_. The surrogate ***s***_*tmp*_(***x***) is assembled from the dataset {***x***_*B*_^(*k*)^,***R***(***x***_*B*_^(*k*)^)}_*k* = 1, …, *NiNd*_. As before, ***R***(***x***_*B*_^(*k*)^) stands for the aggregated circuit characteristics obtained by means of EM analysis. The surrogate ***s***_*tmp*_ is continuously enhanced by inserting the infill points allocated by maximizing the predicted mean square error (MSE) of the current model

11 $${\mathbf{x}}_{B}^{{({N_i}{N_d}+j)}}=\arg \mathop {{\max} }\limits_{{{\mathbf{x}} \in {X_d}}} MSE({{\mathbf{s}}_{tmp}}({\mathbf{x}}))$$

for *j* = 1, 2, …. The model refinement utilizes the extended set {***x***_*B*_^(*k*)^,***R***(***x***_*B*_^(*k*)^)}_*k* = 1, …, *NiNd* + *j*_. The process continues until any of the following termination criteria has been fulfilled:

- Relative RMS error estimated through cross validation^111^ falls below the user-defined threshold *E*_max_, or.
- The total number of samples exceeds 2*N*_*i*_*N*_*d*_ (maximum computational budget).

**Fig. 9:**
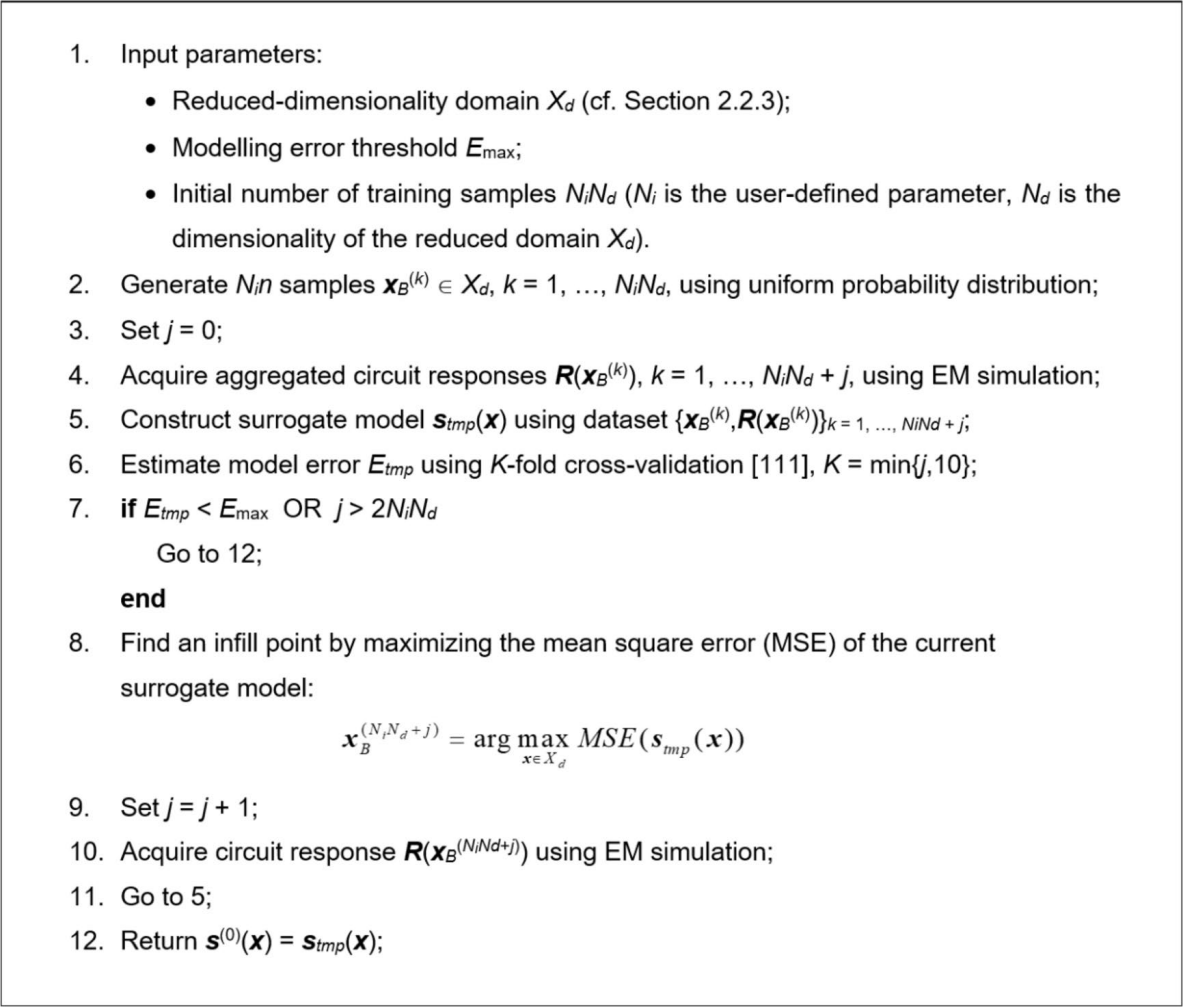
Initial surrogate model construction: the pseudocode.

Maximization of MSE places new training samples at locations corresponding to the maximum predicted error, enhancing the model’s global predictive power within *X*_*d*_. Upon termination, the current model ***s***_*tmp*_(***x***) becomes the first surrogate ***s***^(0)^(***x***). Figure 9 shows a pseudocode of the initial metamodel construction process.

#### Global optimization by machine learning

As mentioned earlier, global search is executed in the reduced domain *X*_*d*_. It is arranged as a surrogate-assisted machine learning framework. The initial predictor is the surrogate model ***s***^(0)^ generated according to a description in Sect. 2.3.1. Further surrogate models, denoted as ***s***^(*j*)^, *j* = 1, 2, …, are produced using the EM data gathered along the optimization path.

Subsequent parameter vectors (approximations of the optimum) ***x***^(*i*+1)^, *i* = 0, 1, 2, …, are produced by (globally) minimizing the merit function *U*_S_(***x***,***s***^(*i*)^(***x***)), which is the same as the function *U* of Sect. 2.1; however, it is evaluated using the current surrogate model ***s***^(*i*)^(***x***) instead of EM simulation. To emphasize that, *U*_*S*_ is marked as explicitly dependent on ***s***^(*i*)^. We have

12 $${x^{(i+1)}}=\arg \mathop {{\min} }\limits_{{x \in {X_d}}} {U_s}\left( {x,{s^{(i)}}\left( x \right)} \right)$$

The sub-task (12) is solved with the help of bio-inspired metaheuristics, specifically, PSO^112^. Note that the underlying objective function *U*_*S*_(***x***,***s***^(*i*)^(***x***)) is cheap to evaluate, the specific selection of the global optimization algorithm is of minor significance: even with relatively large computational budget (e.g., 10,000 function calls), the CPU cost of finding ***x***^(*i*+1)^ can be neglected when juxtaposed against even a single EM simulation of the system at hand. PSO has been selected due to being one of the most recognized algorithms of its class.

From the machine learning perspective, formulating the design task as in (12) is equivalent to using predicted merit function minimization^113^ as the criterion for generating the candidate parameter vectors. The data points ***x***^(*i*)^, *i* = 1, 2, …, approximate the optimum design but are also used (along with the corresponding circuit responses evaluated using EM simulation) to refine the surrogate model. The dataset employed to build ***s***^(*i*)^(***x***) is {***x***_*B*_^(*k*)^,***R***(***x***_*B*_^(*k*)^)}_*k* = 1, …, 2*NiNd* + *i*_, with ***x***_*B*_^(2*NiNd*+*i*)^ = ***x***^(*i*)^ for *i* = 1, 2, ….

Termination of the global optimization phase occurs when one of the two conditions has been satisfied: (i) convergence in argument ||***x***^(*i*+1)^ – ***x***^(*i*)^|| < *ε*, (ii) the lack of the cost function improvement over the last *N*_*no_improve*_ iterations. Both *ε* and *N*_*no_improve*_ are control parameters. Their default values, employed for demonstration case studies of Sect. 3, are *ε* = 10^–2^ and *N*_*no_improve*_ = 20.

### Optimization stage II: local parameter tuning

The global optimization stage described in Sect. 2.3 takes place in the reduced-dimensionality domain *X*_*d*_. Despite the fact that—by definition—*X*_*d*_ accounts for the most part of the circuit output variations, the genuine optimum parameter vector is likely to be allocated outside *X*_*d*_. Consequently, further tuning might be necessary over the complete design variable space *X*, which is realized here using fast gradient-based optimization.

**Fig. 10:**
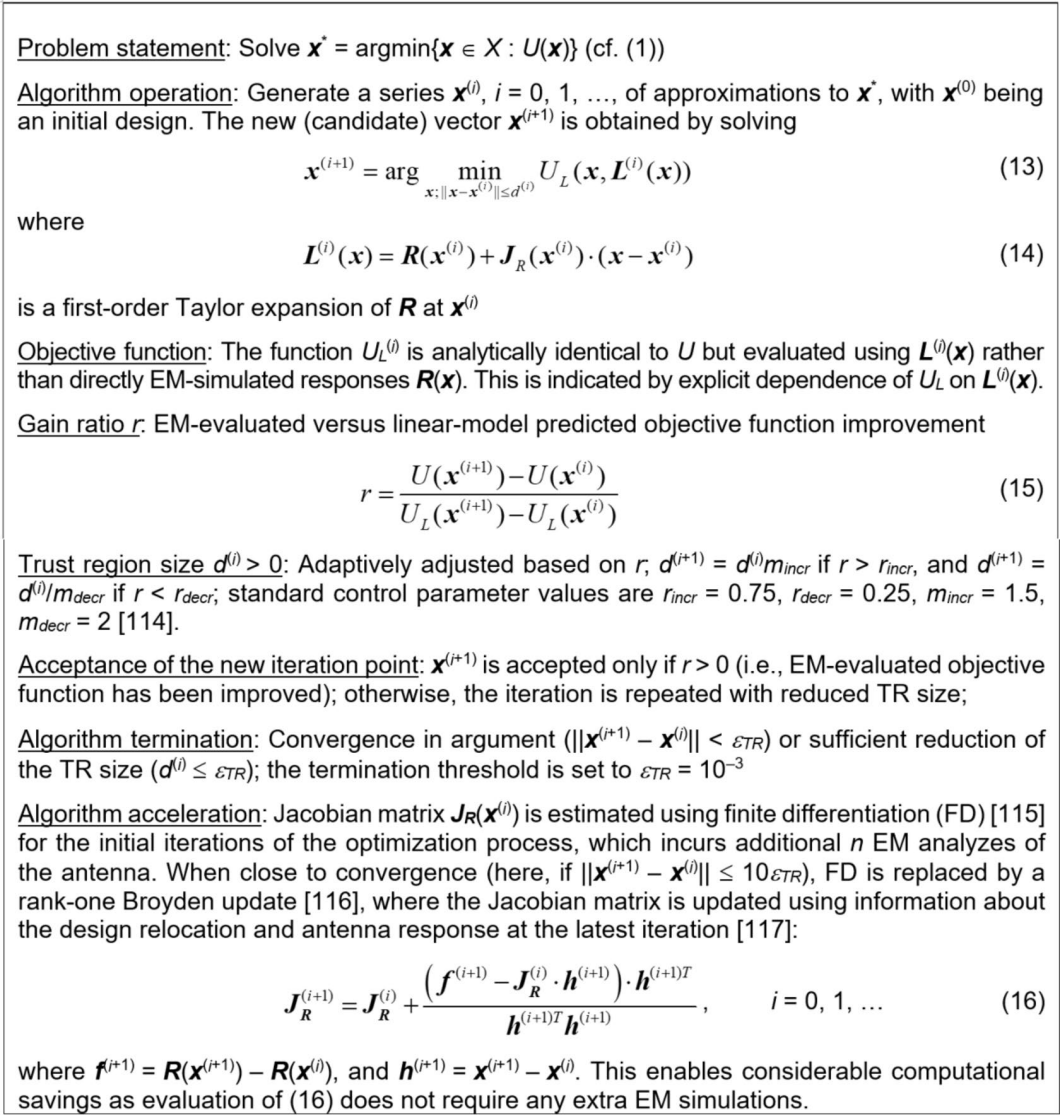
Accelerated trust-region (TR) procedure with derivatives estimated using finite differentiation, and Broyden formula for sensitivity matrix updating.

The gradient-based routine is the core algorithm for fine-tuning the circuit^114^. The procedure has been elucidated in Fig. 10. The algorithm works iteratively by optimizing a local linear representation of the circuit frequency characteristics set up at the most recent design. The search region size is adaptively adjusted by assessing the local model reliability in a given iteration. When closer to convergence, finite differentiation used to compute the sensitivities is substituted by a rank-one Broyden formula. The latter translates to a sizable enhancement of the algorithm’s computational efficiency.

###  Complete procedure

This section explains the overall optimization framework, the building blocks of which have been elaborated on in Sect. 2.2 and 2.3. The main ones are (i) fast global sensitivity analysis (FGSA), (ii) the initial kriging surrogate, (iii) the surrogate-based machine learning scheme, and (iv) trust-region-embedded fine tuning of the system parameters.

Table 1 puts together the control parameters. These were already discussed in the respective sections of the paper. Here, we would like to stress that apart from the parameters controlling the resolution of the search process (*ε*, *N*_*no_improve*_, *ε*_*TR*_), there are only three additional coefficients: *N*_*r*_, *N*_*i*_, and *E*_max_. It is important that none of them is critical. For example, the number of random observables allocated to execute FGSA does not have a significant effect on the sensitivity analysis outcome because the impact of particular parameter space directions is averaged across the entire space. Similarly, both *N*_*i*_ and *E*_max_ are only used for initial surrogate model rendition, which is subsequently refined by means of the machine learning process. Accordingly, there is no need for custom-tuning for a specific optimization problem being solved. To emphasize this point, all demonstration experiments discussed in Sect. 3 utilize identical setup of default control parameter values (cf. the last column of Table 1).

**Table 1:**
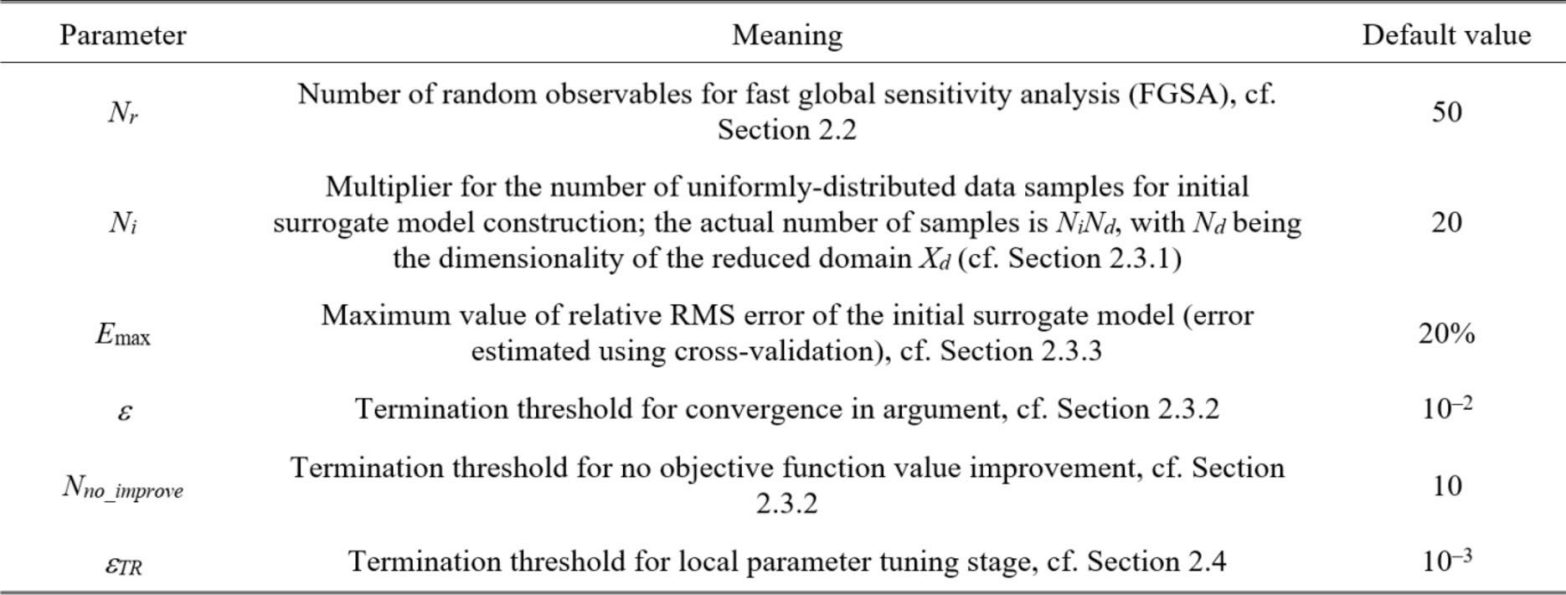
Proposed global optimization framework: control parameters.

**Fig. 11:**
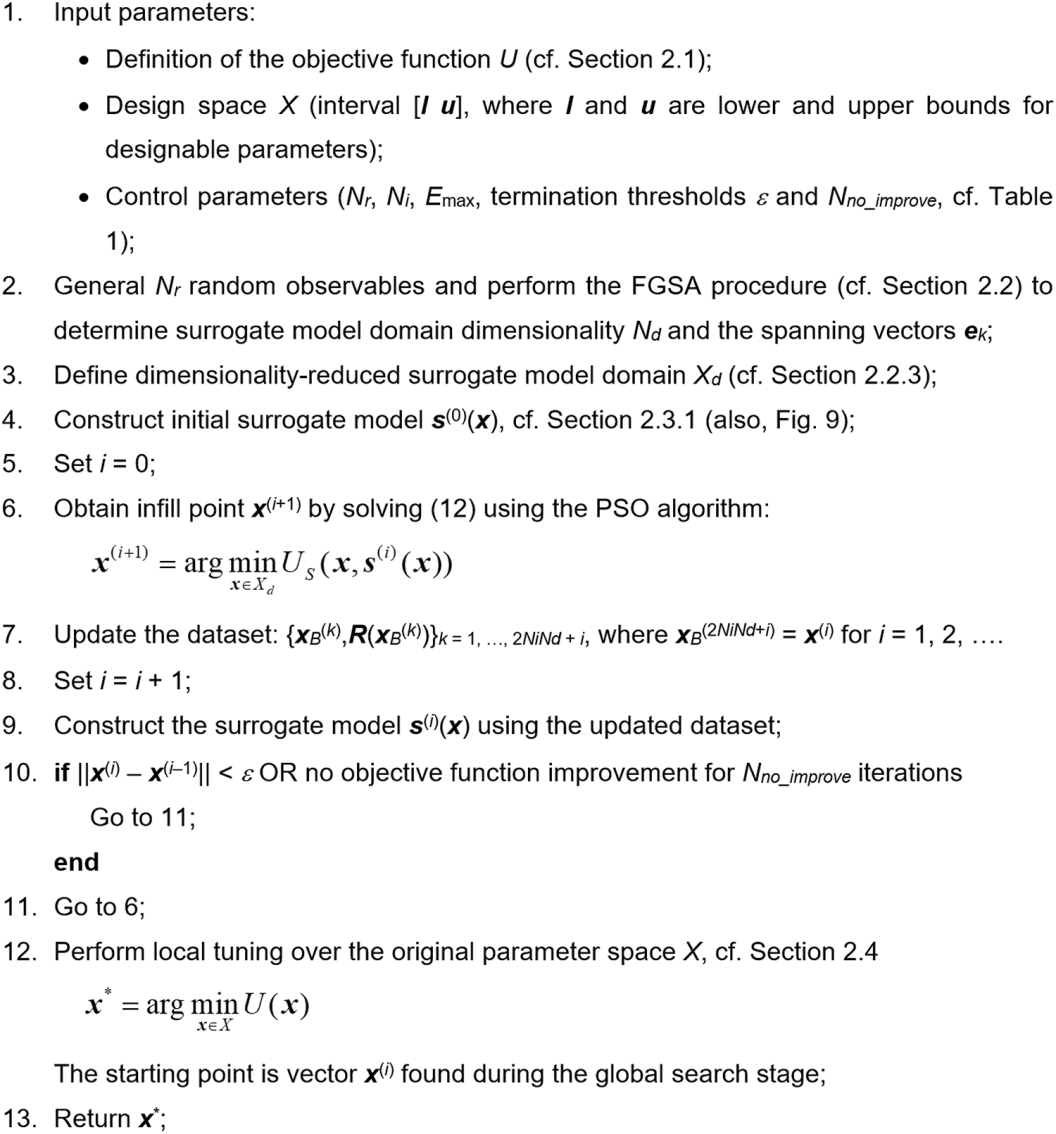
Pseudocode of the ML procedure for globally optimizing passive microwave circuits. The procedure capitalizes on dimensionality-reduced surrogate model, the domain of which is established using fast global sensitivity analysis.

**Fig. 12:**
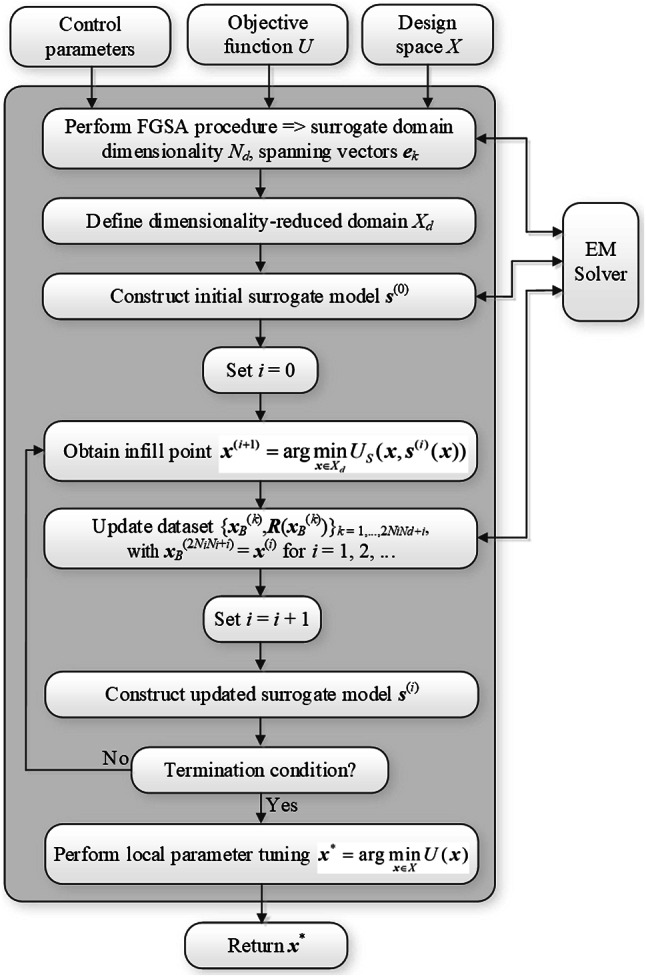
Flow diagram of the FGSA-based machine-learning procedure for globally optimizing microwave circuits.

Figure 11 provides the pseudocode of the suggested surrogate-assisted procedure. As indicated therein, the major stages of the optimization process include global sensitivity analysis (Step 2), the definition of the dimensionality-reduced domain (Step 3), a rendition of the initial surrogate (Step 4), global search using machine learning (Steps 6 through 10), as well as fine-tuning using gradient-based routine (Step 12). The surrogate model is gradually refined within the global optimization phase, whereas local tuning is conducted directly using EM simulations. For supplementary clarification, Fig. 12 illustrates the framework’s flow diagram.

##  Results

The machine learning framework discussed in Sect. 2 is applied to perform global parameter tuning of four microstrip circuits for validation. The test set includes three compact couplers, and a power divider. For all cases, several characteristics are optimized to improve the circuit impedance matching and port isolation and to make sure that the circuit provide equal power division at the target frequencies. The numerical results obtained using our technique are compared to several benchmark methods: nature-inspired optimization using the PSO algorithm, gradient-based search initiated from random starting points, and machine learning procedure operating in the original design variable space *X*. The algorithm performance is assessed based on several indicators that include the design quality, the ability of the algorithm to identify the design satisfying the specifications (e.g., to align its operating frequency with the target one), along with the CPU cost of the algorithm.

The remaining sub-sections are arranged as follows. The verification circuits are introduced in Sect. 3.1. Section 3.2 provides the details of the experimental setup and the numerical results. Section 3.3 discusses the findings, and compares our framework to the benchmark methods.

### Test cases

The algorithm verification is based on four microstrip circuits, as listed below:

- Miniaturized rat-race coupler with meandered TLs (Circuit I)^118^;
- Compact branch-line coupler with CMRCs (Circuit II)^119^;
- Compact branch-line coupler with CMRCs (Circuit III)^120^;
- Dual-band power divider (Circuit IV)^121^.

**Fig. 13:**
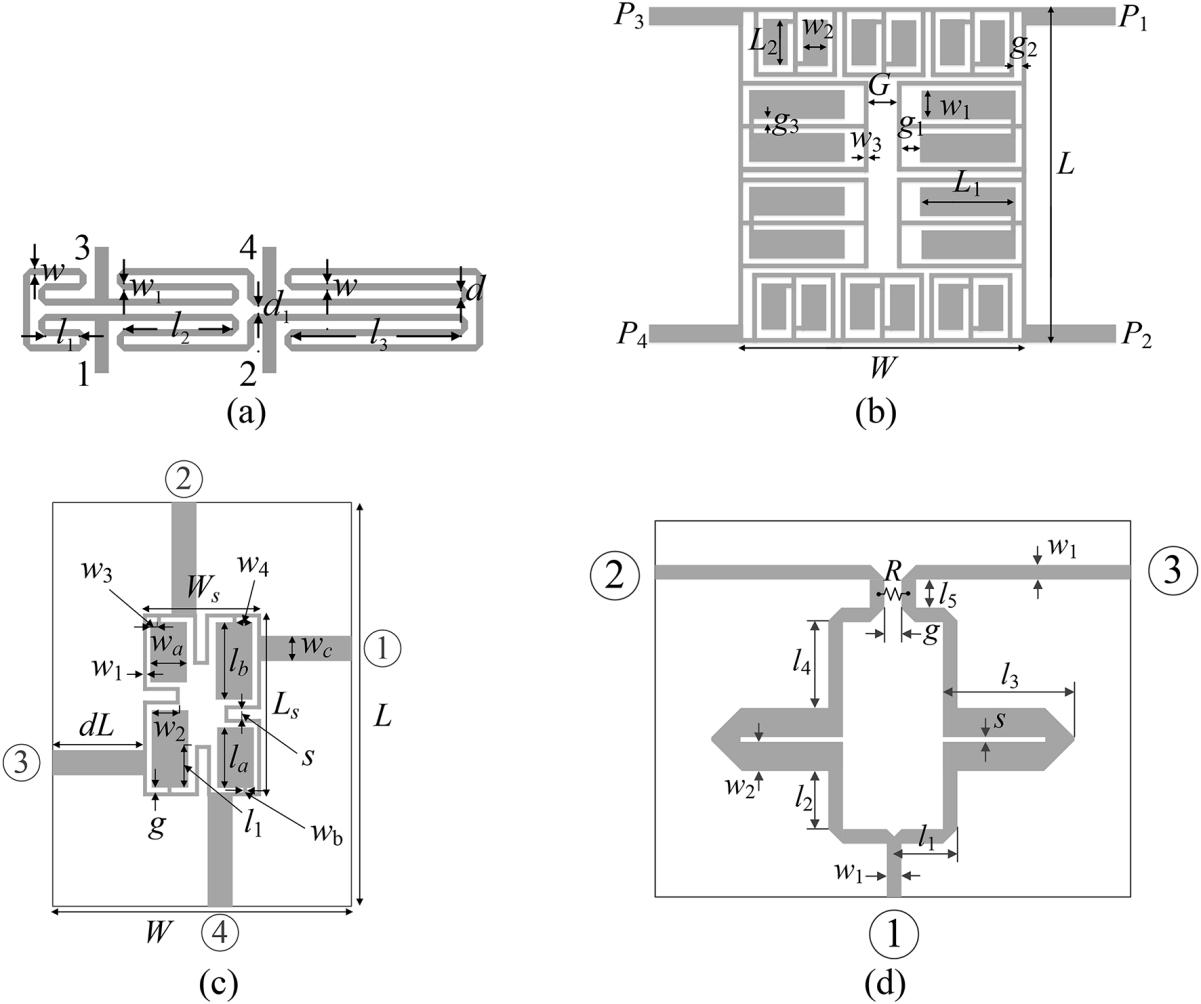
Verification circuits: (**a**) Circuit I^118^, (**b**) Circuit II^119^, (**c**) Circuit III^120^, (**d**) Circuit IV^121^.

Figure 13 shows the circuit geometries. The essential information concerning the material parameters, design variables, lower/upper bounds for design variables defining the original space *X*, and specific design tasks are included in Table 2. EM models of all structures are implemented in CST^122^. The design objectives for the coupling circuits are to enhance impedance matching and port isolation responses and maintain an equal power division ratio at the target center frequency. For the last circuit, the goal is to improve return loss and port isolation at all ports, maintaining equal power split at both center frequencies *f*_1_ and *f*_2_. In this structure, however, the latter property is automatically maintained due to the circuit symmetry, therefore, it is not explicitly incorporated into the cost function.

Note that the considered verification problems are challenging due to several factors: (i) high nonlinearity of the circuit characteristics, (ii) relatively high dimensionality (from six for Circuit I to ten for Circuit III), (iii) large parameter spaces (the average upper-to-lower ratio of the design variable bounds is 13, 5, 2, and 12 for Circuit I through IV, accordingly), (iv) the necessity of simultaneous handling of several responses (typically four per circuit, e.g., *S*_*k*1_, *k* = 1, 2, 3, 4, for couplers).

**Table 2:**
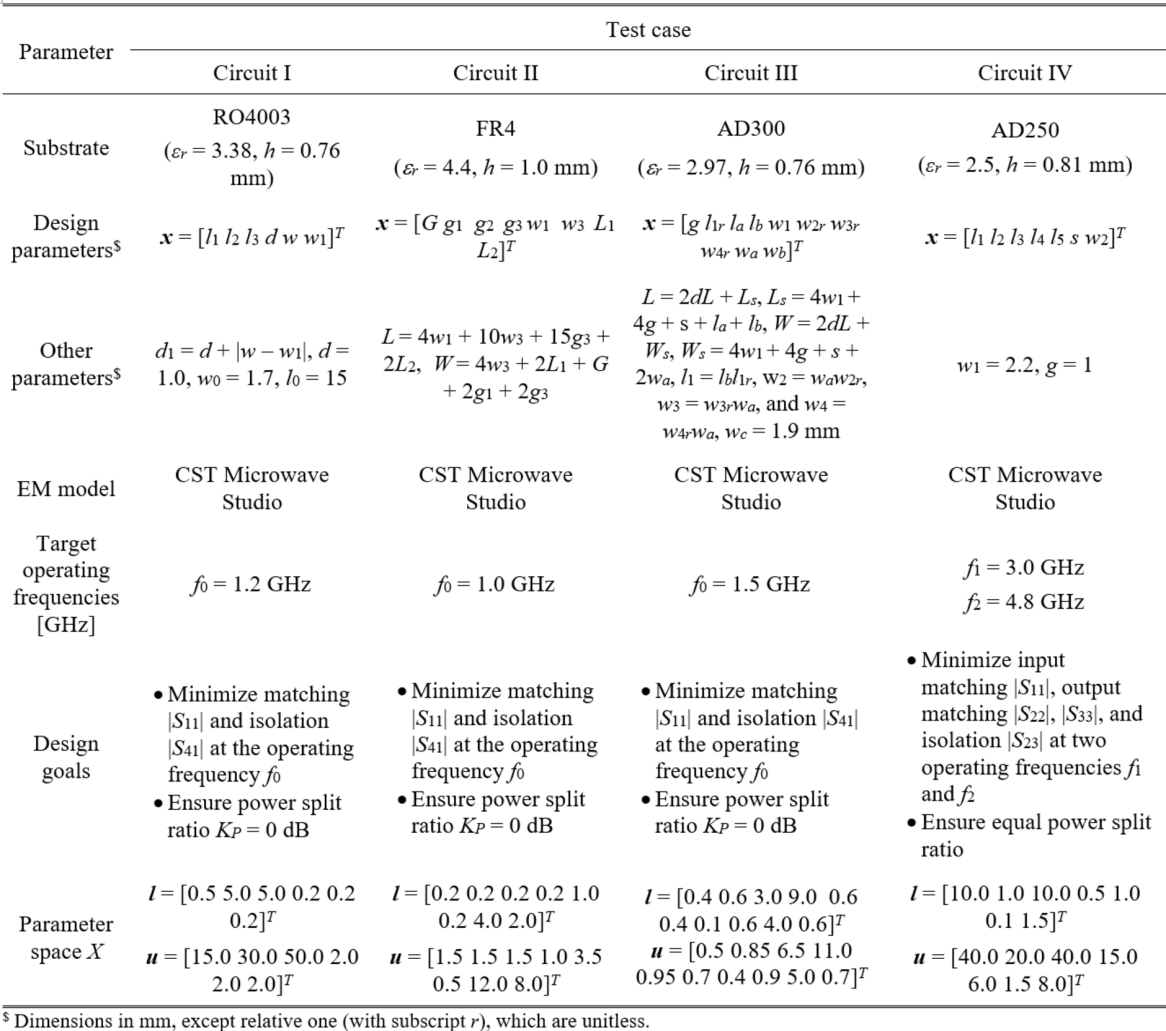
Verification circuits: essential data and design specifications.

### Setup and results

The arrangement of the suggested procedure can be found in Table 3. Note that all control parameters are set at their default values as indicated in Table 1. The setup remains unchanged for all four test problems in order to demonstrate that no extra tuning is required for any specific task at hand. Table 3 also provides the setup details of the benchmark methods, which are the following:

- Algorithm I: PSO^112^, employed as probably the most widely used bio-inspired procedure. PSO is executed in two versions, with the computational budget of 500 (Version I) and 1,000 (Version II) objective function evaluations. While this budget is low for population-based methods, it is significant in the realm of EM-based design. It is intentionally kept at the mentioned numbers to ensure meaningful comparison with other methods.
- Algorithm II: multiple-start gradient search, with the core search procedure being the TR routine (cf. Section 2.4). This method is used to corroborate that the verification problems tasks are multimodal. In particular, multiple local optima should make the algorithm converge to solutions that are away from the global optimum.
- Algorithm III: a surrogate-based machine-learning procedure using kriging and predicted improvement of the merit function as an infill criterion. The algorithm operates within the original parameter space *X*. It is incorporated into the benchmark set to show the advantages of FGSA-based dimensionality reduction as proposed in Sect. 2.

**Table 3:**
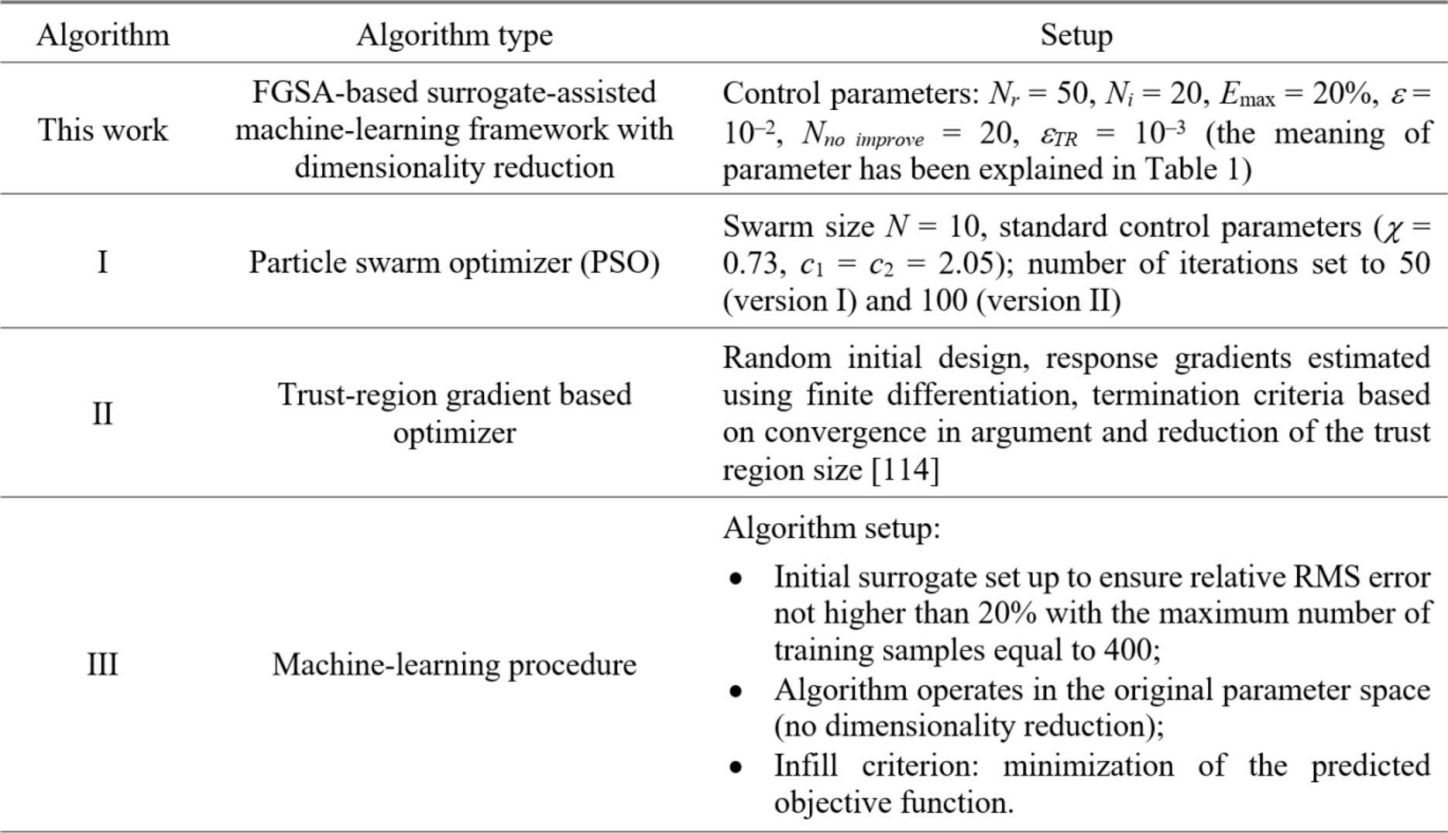
Proposed and benchmark algorithm setup.

Tables 4, 5, 6 and 7 contain the numerical results. Due to the stochastic nature of the considered techniques (both proposed and benchmark), each algorithm has been executed ten times. The data included in the tables stand for the averages of the performance indicators, namely, the merit function value, and the CPU expenses. Furthermore, we also report a so-called success rate, representing the number of algorithm runs that led to satisfactory results (here, achieving good agreement of the center frequencies with their intended values).

It should be emphasized that the computational cost of the optimization process is expressed in terms of the number of full-wave electromagnetic (EM) simulations of the respective circuit, which is a meaningful way of comparing the efficiency of the proposed and the benchmark methods. This is because the EM analysis time contributes to the vast majority of the overall CPU expenses. For the considered circuits, the typical (individual) simulation time is between two and five minutes of the CPU time, meaning that the cost of the entire optimization process amounts to a few hours (for the proposed technique) and up to a few days for the most expensive benchmark routines. All other costs (e.g., associated with the construction of the surrogate model) are negligible (for example, identification of a kriging interpolation model takes around one second for the typical number of training samples, here, up to a few hundred). Consequently, a comparison of the number of EM analyses entailed by each method gives a complete and precise account of their efficiency. In terms of memory consumption, again, the vast majority of it is associated with full-wave EM simulation using commercial solvers (here, CST Microwave Studio). The specific amount of memory is difficult to estimate and it is typically a few GB or RAM, depending on the circuit under design. Most of the memory is actually taken by the solver front-end, not the simulation process itself. The memory required by all other operations (e.g., surrogate identification and core algorithm operations) is negligible.

**Fig. 14:**
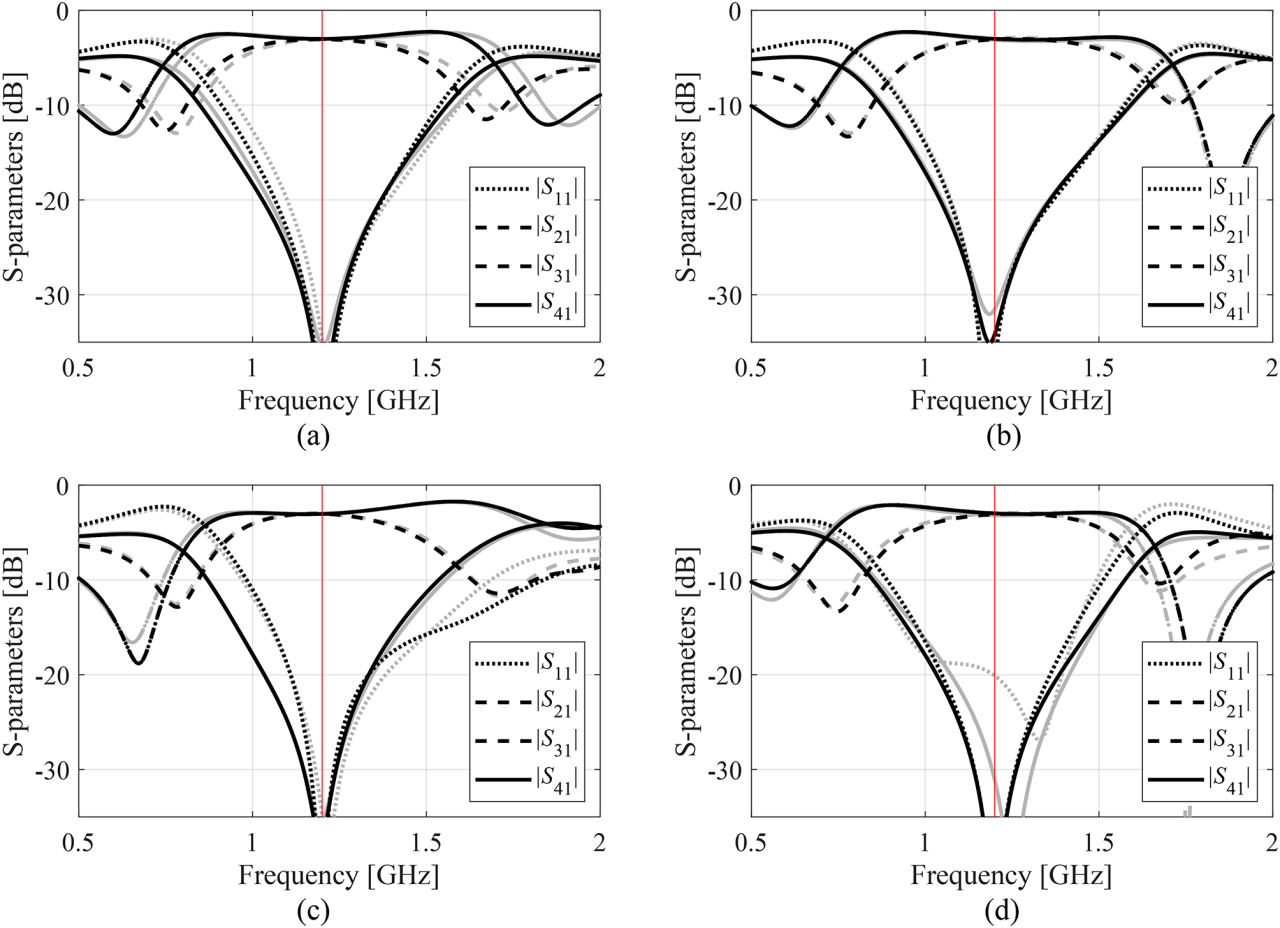
Responses of Circuit I at the optimized designs obtained using the suggested surrogate-assisted machine learning procedure for representative runs: run 1 through 4 (a)-(d). Gray lines stand for the outputs at the starting point ***x***^(0)^ generated by the global phase; black lines correspond to the characteristics at the optimized design. The vertical line marks the target center frequency, here 1.2 GHz.

**Fig. 15:**
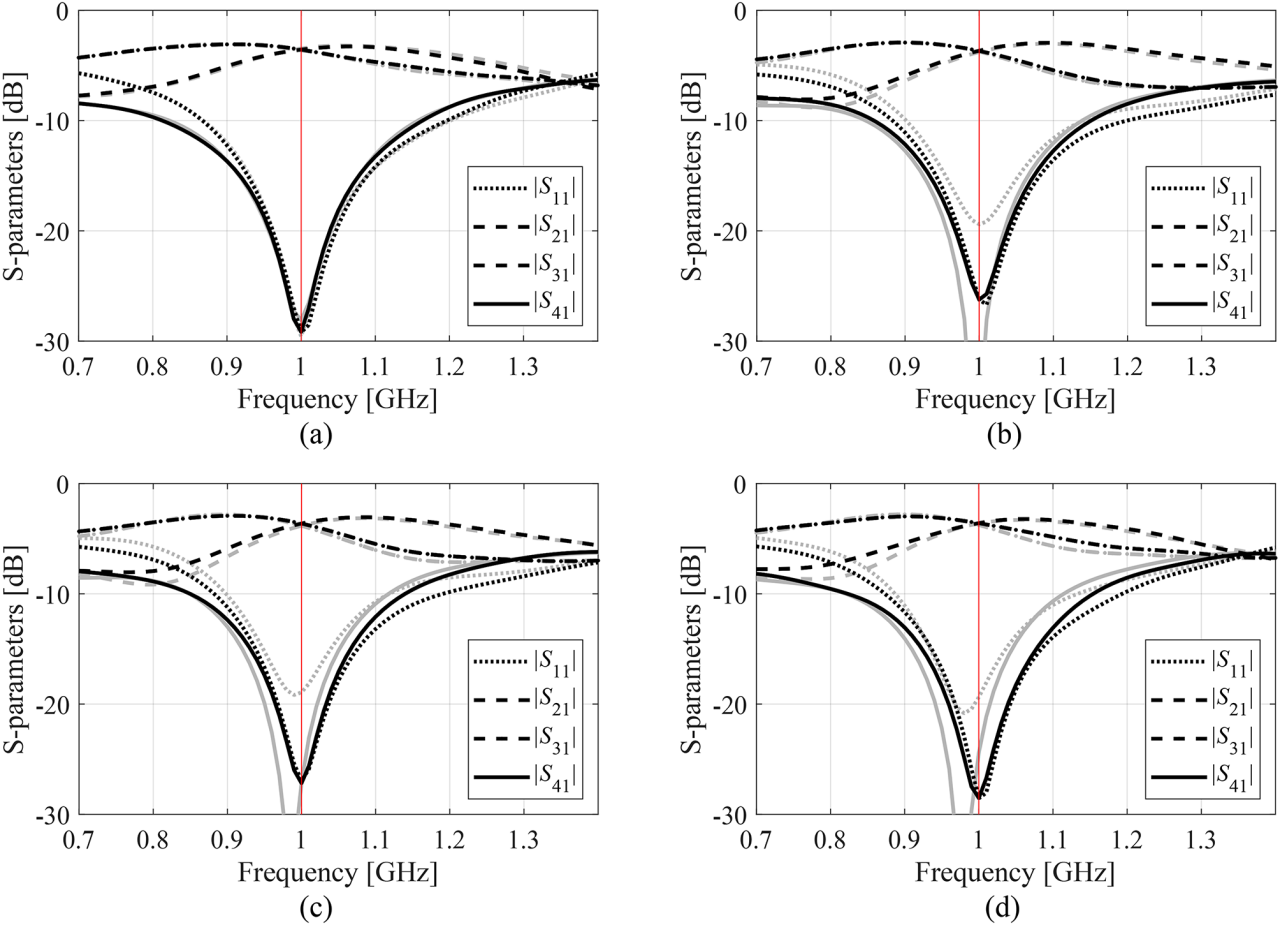
Responses of Circuit II at the optimized designs obtained using the suggested surrogate-assisted machine learning procedure for representative runs: run 1 through 4 (a)-(d). Gray lines stand for the outputs at the starting point ***x***^(0)^ generated by the global phase; black lines correspond to the characteristics at the optimized design. The vertical line marks the target center frequency, here 1.0 GHz.

**Fig. 16:**
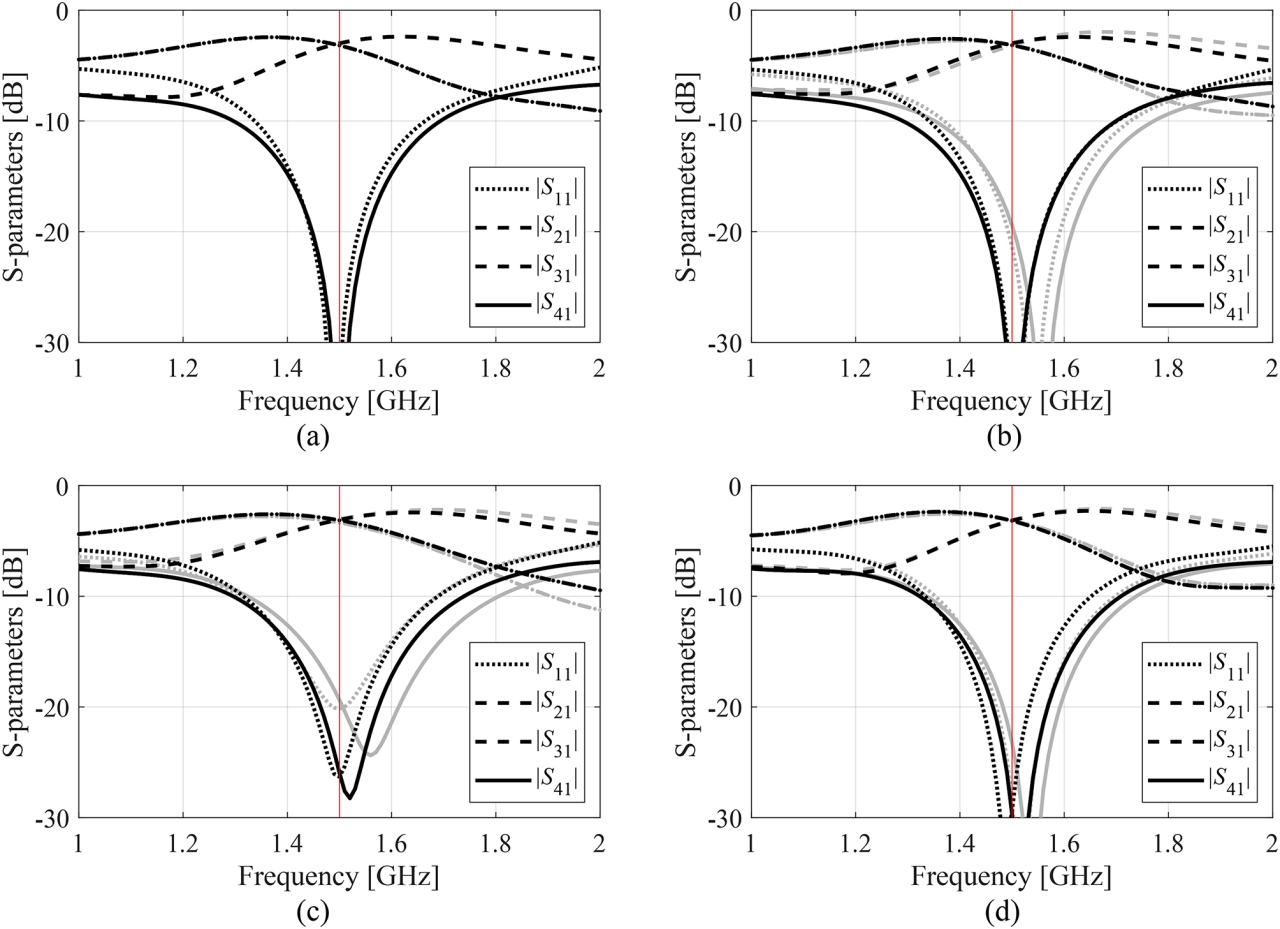
Responses of Circuit III at the optimized designs obtained using the suggested surrogate-assisted machine learning procedure for representative runs: run 1 through 4 (a)-(d). Gray lines stand for the outputs at the starting point ***x***^(0)^ generated by the global phase; black lines correspond to the characteristics at the optimized design. The vertical line marks the target center frequency, here 1.5 GHz.

**Fig. 17:**
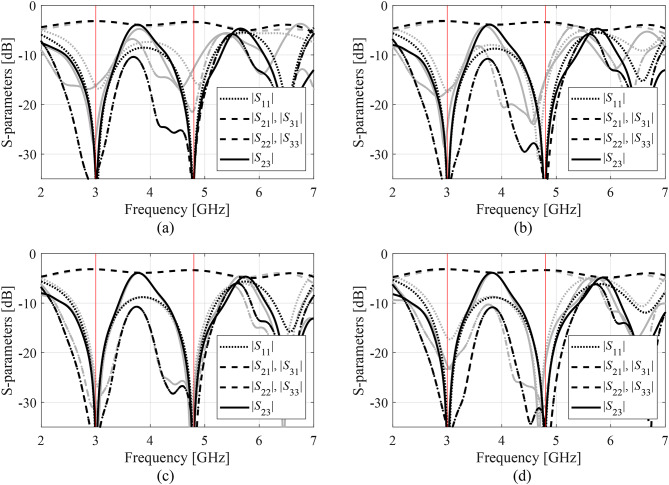
Responses of Circuit IV at the optimized designs obtained using the suggested surrogate-assisted machine learning procedure for representative runs: run 1 through 4 (a)-(d). Gray lines stand for the outputs at the starting point ***x***^(0)^ generated by the global phase; black lines correspond to the characteristics at the optimized design. Vertical lines mark the target operating frequencies, here 3.0 GHz and 4.8 GHz.

Figures 14, 15 and 16, and 17 show the frequency responses of the Circuits I through IV, for the representative algorithm executions. The pictures illustrate the responses obtained upon accomplishing the global search phase and the circuit outputs at the optimized designs.

**Table 4:** Optimization results for circuit I.

Optimization algorithm	Performance figure		
	Average objective function value [dB]	Computational cost^$^	Success rate^#^
Algorithm I: PSO (50 iterations)	–24.8	500	9/10
Algorithm I: PSO (100 iterations)	–34.0	1,000	10/10
Algorithm II: Trust-region gradient-based algorithm	–18.7	102.8	6/10
Algorithm III: Machine learning operating in the original parameter space *X*	–34.1	435.7	10/10
Proposed algorithm	–38.6	155.0	10/10

^$^The cost expressed in terms of the number of EM simulations of the circuit under design.

^#^Number of algorithm runs at which the operating frequencies were allocated in the vicinity of the target frequency.

**Table 5:** Optimization results for circuit II.

Optimization algorithm	Performance figure		
	Average objective function value [dB]	Computational cost^$^	Success rate^#^
Algorithm I: PSO (50 iterations)	–20.8	500	9/10
Algorithm I: PSO (100 iterations)	–22.2	1,000	9/10
Algorithm II: Trust-region gradient-based algorithm	–7.5	49.0	5/10
Algorithm III: Machine learning operating in the original parameter space *X*	–26.2	449.8	10/10
Proposed algorithm	–24.5	183.6	10/10

^$^The cost expressed in terms of the number of EM simulations of the circuit under design.

^#^Number of algorithm runs at which the operating frequencies were allocated in the vicinity of the target frequency.

**Table 6:** Optimization results for Circuit III.

Optimization algorithm	Performance figure		
	Average objective function value [dB]	Computational cost^$^	Success rate^#^
Algorithm I: PSO (50 iterations)	–25.2	500	9/10
Algorithm I: PSO (100 iterations)	–29.1	1,000	9/10
Algorithm II: Trust-region gradient-based algorithm	–10.7	57.4	8/10
Algorithm III: Machine learning operating in the original parameter space *X*	–30.2	238.4	10/10
Proposed algorithm	–25.7	165.4	10/10

^$^The cost expressed in terms of the number of EM simulations of the circuit under design.

^#^Number of algorithm runs at which the operating frequencies were allocated in the vicinity of the target frequency.

**Table 7:** Optimization results for Circuit IV.

Optimization algorithm	Performance figure		
	Average objective function value [dB]	Computational cost^$^	Success rate^#^
Algorithm I: PSO (50 iterations)	–23.7	500	9/10
Algorithm I: PSO (100 iterations)	–36.2	1,000	10/10
Algorithm II: Trust-region gradient-based algorithm	48.3	68.7	5/10
Algorithm III: Machine learning operating in the original parameter space *X*	–45.6	433.6	10/10
Proposed algorithm	–42.5	282.2	10/10

^$^The cost expressed in terms of the number of EM simulations of the circuit under design.

^#^Number of algorithm runs at which the operating frequencies were allocated in the vicinity of the target frequencies.

### Discussion

Here, we summarize the outcome of verification studies, discuss the performance of the introduced method, and compare it to the benchmark algorithms. The discussion is arranged with respect to the main performance indicators considered in Tables 4, 5, 6 and 7.

#### Global search capability and optimization process reliability

The proposed procedure performs consistently for all four circuits with the perfect success rate of 10/10. This corroborates its ability to find a satisfactory result in each and every run. A low success rate for Algorithm II (only 6/10, 5/10, 8/10, and 5/10 for Circuit I through IV, respectively) confirms multimodality of the considered design problems. The performance of Algorithm I (PSO) is considerably better. Its success rate is close to perfect, although it is evident—by comparing the results obtained for 500 and 1,000 objective function calls—that neither the budget of 500 nor 1,000 is sufficient. This is to be expected as typical costs of nature-inspired algorithms are measured in thousands of objective function evaluations. Here, it has been restricted for practical reasons, although even then, the typical running time is two to four days (problem dependent). Algorithm III (the ML procedure) demonstrated the best reliability, which is comparable to that of the method presented in this work (the success rate of 10/10); however, because of operating in the original parameter space, its computational efficiency is inferior.

#### Design quality

The quality of the outcomes produced using our methodology, assessed by the cost function values, is comparable to that obtained with other global search procedures, including PSO (Algorithm I), and machine learning operating in the full-dimensionality space (Algorithm III). For some of the circuits, the results are slightly better; however, the practical differences of a few dB are minor given that the typical objective function values are at the levels of − 30 dB or below. This means, in particular, that dimensionality reduction does not jeopardize the reliability of the algorithm.

#### Computational efficiency

Among the considered global search frameworks, the efficacy of our approach is by far the best. The average expenses associated with the optimization process correspond to less than 200 EM analyses, which is almost 50% less than the expenses entailed by the machine learning procedure operating within the original (full-dimensionality) parameter space. The savings are mainly due to a considerably lower expenses at the surrogate modeling stage, and are achieved despite additional costs related to the sensitivity analysis and final tuning. At the same time, the computational savings over nature-inspired optimization (the version with 1,000 merit function calls set as the algorithm’s budget) are as high as 80%.

The results obtained for the presented verification case studies demonstrate that the proposed surrogate-assisted machine learning framework does exhibit global search capability. Its performance is consistent across all considered circuits with good solution repeatability. Furthermore, our algorithm yields designs of excellent quality as compared to the benchmark, and shows competitive computational efficiency. These merits are directly related to the employment of fast global sensitivity analysis, leading to a restriction of the search space dimensionality. This reduction enables the construction of reasonable-quality surrogate models at a fraction of the cost needed when building the model in the original design variable space, which carried over to improved efficacy of the optimization procedure. Another important benefit of the discussed approach is a limited number of control parameters. Half of these (termination-related coefficients) decide upon the search process resolution. The remaining three parameters are not critical for the performance due to self-adjustment mechanisms implemented therein. This feature has been demonstrated by running all verification experiments using identical control parameter setup.

It should be emphasized that as the methodology considered in the manuscript is an optimization algorithm (not a modeling framework), the specific predictive power of the surrogates is irrelevant. This is because, at each design stage, the design quality produced by the algorithm is continuously assessed through full-wave EM analysis. The surrogate model is just an aid. Its accuracy changes from iteration to iteration as the model is refined during the optimization run, using the acquired EM data. Also, when focusing on the promising regions of the design variable space, its predictive power improves over this region while remaining inferior everywhere else. The results in Tables 4, 5, 6 and 7 and in Figs. 14, 15, 16 and 17 are all based on EM simulations. Consequently, the accuracy of the surrogate leading to these results is, again, irrelevant.

## Conclusion

This research focused on developing an innovative approach to global parametric optimization of passive circuits. The presented methodology leverages reduction of the dimensionality of the parameter space, which is achieved using a custom-developed fast global sensitivity analysis (FGSA). FGSA enables the determination of directions having the main effects on the circuit outputs. The global search stage is limited to the affine subspace constructed using a small subset of the most influential vectors among those determined by the sensitivity analysis. The said subspace becomes a domain for a kriging metamodel established to predict the spatial allocation of the optimum parameter vector within the machine learning (ML) framework. ML employs predicted merit function enhancement for generating candidate designs. Global search is supplemented by local parameter tuning conducted over the entire (not reduced) parameter space. The optimization framework incorporating the mentioned algorithmic components has been demonstrated to operate reliably and in a consistent manner for a large pool of verification case studies. Extensive comparisons with several state-of-the-art routines, including a bio-inspired algorithm and an ML scheme operating within the original parameter space, indicate competitive efficacy regarding the quality of the rendered designs and CPU efficiency. The CPU savings achieved due to dimensionality reduction are as high as 50%. Other advantages of the presented framework include its generality (no assumptions are made upon the circuit structure or its characteristics), straightforward setup being a consequence of a small set of control parameters, as well as straightforward and modular implementation. These features contribute to making the proposed technique a potentially attractive methodology compared to the global search techniques available in the literature and used in engineering practice.

## Data Availability

The datasets used and/or analyzed during the current study available from the corresponding author on reasonable request.
